# Human endogenous retrovirus K (HML-2) RNA and protein expression is a marker for human embryonic and induced pluripotent stem cells

**DOI:** 10.1186/1742-4690-10-115

**Published:** 2013-10-24

**Authors:** Nina V Fuchs, Sabine Loewer, George Q Daley, Zsuzsanna Izsvák, Johannes Löwer, Roswitha Löwer

**Affiliations:** 1 Paul-Ehrlich-Institute, Federal Institute for Vaccines and Biomedicines, Paul-Ehrlich-Str. 51-59, D- 63225 Langen, Germany; 2 Division of Pediatric Hematology and Oncology, Children’s Hospital Boston and Department of Biological Chemistry and Molecular Pharmacology, Harvard Medical School, Boston, MA, USA; 3 Present address: Max Delbrück Center for Molecular Medicine, Robert-Rössle-Str. 10, D- 13125 Berlin, Germany; 4 Mobile DNA, Max-Delbrück-Center for Molecular Medicine, Robert-Rössle-Str. 10, D- 13092 Berlin, Germany

**Keywords:** Human embryonic stem cells, Induced pluripotent stem cells, Activation of human endogenous retrovirus K proviruses, Embryoid body differentiation, Pluripotency marker

## Abstract

**Background:**

Malignant human embryonal carcinoma cells (ECCs) rely on similar transcriptional networks as non-malignant embryonic stem cells (ESCs) to control selfrenewal, maintain pluripotency, and inhibit differentiation. Because re-activation of silenced *HERV-K(HML-2)* loci is a hallmark of ECCs, we asked if this HERV group was also reactivated in ESCs and induced pluripotent stem cells (iPSCs).

**Findings:**

Using RT-PCR and Western Blot, we demonstrate *HERV-K(HML-2)* RNA and protein expression in undifferentiated human ESCs and iPSCs. Induction of differentiation by embryoid body formation resulted in rapid silencing of *HERV-K(HML-2)* provirus expression. Sequencing analysis of a conserved region of the *gag* gene showed that proviral expression in ESCs and iPSCs represents at least 11 of the 66 nearly full length *HERV-K(HML-2)* loci, with slightly varying patterns in individual cell lines. These proviruses are human specific integrations and harbor promoter competent long terminal repeats (LTR5hs subgroup). We observed high mRNA levels of the NP9 and Gag encoding proviruses *K101(22q11.21)* in all and *K10(5q33.3)* in most of the ECC, ESC, and iPSC lines tested, while *K37(11q23.3)* mRNA was detected only in ESCs and iPSCs. In addition, we detected expression of proviral mRNA encoding the RNA export adaptor Rec in all cell lines studied. Proviral mRNA originating from the *K108(7p22.1)* locus, which inter alia codes for functional Rec and Env proteins, was only reactivated in malignant ECC lines, not in benign ESCs or iPSCs.

**Conclusions:**

*HERV-K(HML-2)* RNA and protein expression is a marker for pluripotent human stem cells. Initiation of differentiation results in rapid down-regulation. Further studies are needed to explore a putative functional role of HERV-K(HML-2) RNA and proteins in pluripotent stem cells.

## Findings

**Figure 1:**
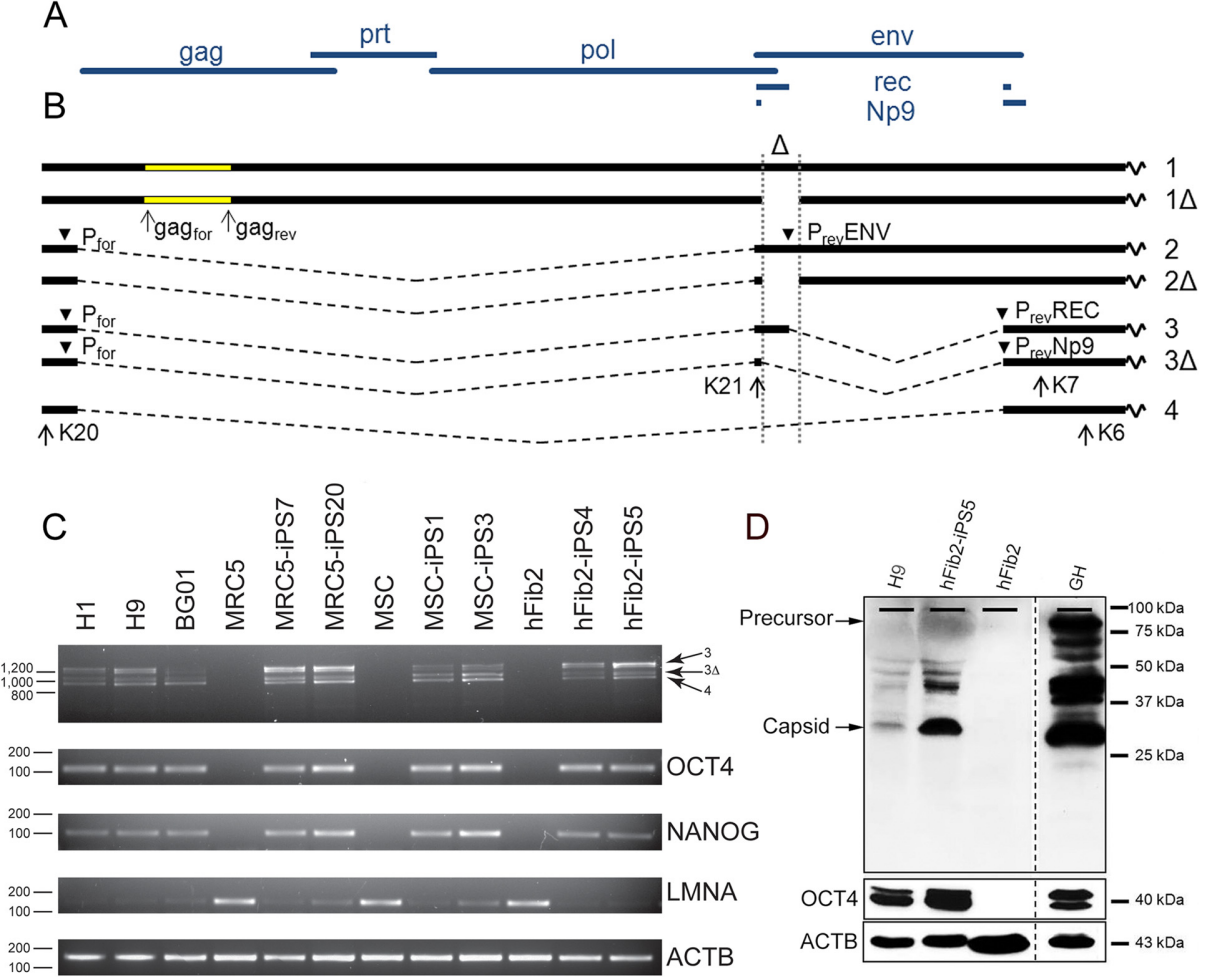
***HERV-K(HML-2) *expressed at RNA and protein level in ES/iPS cells. A)** Prototypic HERV-K(HML-2) open reading frames **B)** Type 1 and 2 mRNA species, ∆ denotes the 292 bp deletion in type 1 proviruses. **1)***full length* type 2, **1**∆**)***full length* type 1, **2)***env* mRNA type 2, **2**∆**)***env* mRNA type 1, **3)** 1.8 kb mRNA type 2 encoding *rec*[18], **3**∆**)** 1.5 kb mRNA type 1 encoding *Np9*[4], **4)** 1.5 kb mRNA derived from type 1 or type 2 [18]. PCR primers are indicated by arrows and arrowheads. Primers P_rev_ENV, P_rev_REC, P_rev_NP9 overlap the immediate upstream splice site. **C)** RT-PCR of *HERV-K(HML-2)* expression in ES cells (H1, H9, BG01), fibroblasts (MRC5, MSC, hFib2) and corresponding iPS cells using the primer pair K20 and K6. *OCT4, NANOG* served as pluripotency, *LMNA* as differentiation markers, *ACTB* as internal control [19,20]. **D)** Western blot for HERV-K(HML-2) Gag, OCT4 and ACTB in ES, iPS, fibroblast and EC cells.

Although no infectious HERV particles have been detected to date, several proviruses of the evolutionary young hominoid group *HERV-K(HML-2)* harbor open reading frames for viral proteins, as well as promoter competent long terminal repeats (LTRs) [1,2]. Two subgroups exist (Figure 1A): Type 2 proviruses encode the accessory protein Rec, which mediates nuclear-cytoplasmic translocation of incompletely spliced RNA, thereby allowing translation of viral proteins [3]. Type 1 proviruses harbor a 292-bp deletion within the pol-env boundary, resulting in loss of a functional open reading frame for Rec, but gaining a functional open reading frame for the accessory protein Np9 [4]. *In vitro* assays have shown that Rec and Np9 interact with cellular proteins like the promyelocytic leukemia zinc finger protein (PLZF), ligand of numb protein X (LNX), testicular zinc-finger protein, androgen receptor, and small glutamine-rich tetratricopeptide repeat protein and might thus support cell transformation [5-9]. Although *HERV-K(HML-2)* proviruses may be transcribed in somatic cells [10,11], up-regulated transcription and protein expression, formation of retrovirus particles, and induction of an anti*-HERV-K(HML-2)* immune response are predominantly associated with germ cell tumors, embryonal carcinoma cell (ECC) lines, melanomas and other cancers [12-17].

The phenotype and transcriptional profile of ECCs resemble that of non-malignant pluripotent embryonic stem cells (ESCs) and induced pluripotent stem cells (iPSCs) in that they express key pluripotency factors such as OCT4, NANOG and SOX2 and other markers characteristic of pluripotent stem cells [reviewed in [21]]. In addition, ECCs rely on similar pathways to regulate self-renewal and inhibition of differentiation [21], such as autocrine FGF signalling and activation of downstream signalling cascades, especially the ERK/MEK pathway [22,23]. These similarities prompted us to test whether *HERV-K(HML-2)* RNA and protein expression, which is a marker of ECCs, might also be reactivated in non-malignant pluripotent stem cells.

### *HERV-K(HML-2)* RNA and proteins are expressed in ESCs and iPSCs

We analyzed the expression of *HERV-K(HML-2)* in three ESC lines (H1, H9, BG01), three fibroblast lines (MRC5, MSC, hFib2) and iPSC lines derived from these fibroblasts. All materials and methods are described in detail in Additional file 1 “Materials and Methods”. To augment the probability to detect *HERV-K(HML-2)* transcripts derived from proviral promoters, we analyzed completely spliced viral RNA by RT-PCR. Full length and spliced transcripts and the location of all primer pairs used are depicted in Figure 1B. We verified expression of the endogenous pluripotency genes *OCT4* and *NANOG* and absence of the differentiation marker gene *LMNA* in pluripotent stem cells*, ACTB* specific amplicons served as internal controls [19,20]. We did not detect any *HERV-K(HML-2)* specific amplicons in any of the fibroblast cell lines, which readily expressed the differentiation marker *LMNA*. In contrast, all ESC and iPSC lines tested showed expression of all three types of completely spliced *HERV-K(HML-2)* transcripts, in addition to the pluripotency markers *OCT4* and *NANOG* (Figure 1C). The intensities of the PCR amplicons varied between individual ESC/iPSC lines, suggesting differential activation of proviruses between lines.

The presence of spliced *HERV-K(HML-2)* type 2 transcripts in all pluripotent stem cell lines tested prompted us to investigate whether they encode functional Rec protein and thus enable translation of *HERV-K(HML-2)* Gag and Env proteins. Using a *HERV-K(HML-2)* Gag specific monoclonal antibody in Western blot analysis, we indeed detected low to moderate levels of this protein in the H9 ESCs and the hFib2-iPS5 iPSC line, but not in the parental fibroblasts hFib2. By contrast, the ECC line GH showed high levels of Gag protein expression (Figure 1D). To ensure the phenotypic similarity between the malignant ECC line and the non-malignant ESC/iPSC lines, we verified by RT-PCR that GH cells expressed the pluripotency markers *OCT4*, *NANOG*, *SOX2*, *STELLA* as well as the autocrine signaling factor *FGF4* (data not shown). The differences in *HERV-K(HML-2)* Gag protein levels observed in H9 and hFib2-iPS5 correlated with the differences in signal intensities of their *rec* specific amplicons in RT-PCRs (Figure 1C, compare intensities of amplicons 3), which is consistent with the idea that the levels of functional Rec protein determine the efficiency of Gag protein translation.

### Differentiation induces silencing of *HERV-K(HML-2)* proviruses

**Figure 2:**
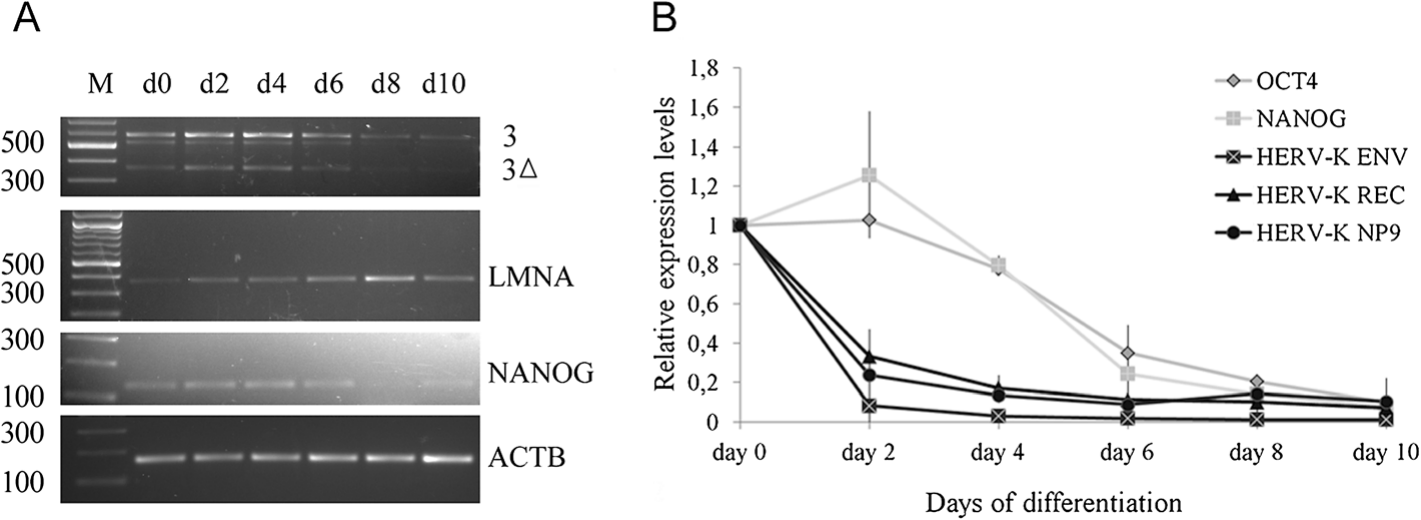
***HERV-K(HML-2) *expression is immediately down-regulated during embryoid body differentiation. A)** Semi-quantitative RT-PCR of *HERV-K(HML-2)* expression in hFib2- iPS5 cells using the primer pair K21 and K7. The faint band at ≈570 bp presumably represents the *rec* mRNA of provirus ERVK-17 (see Table 1) which contains a small deletion in the amplified region. LMNA was amplified using an alternative primer (see Additional file 1 “Materials and Methods”) **B)** Quantitative expression analysis of *HERV-K(HML-2) env*, *rec* and *Np9* transcripts in H9 cells. *OCT4*, *LMNA*, and *ACTB* serve as control [19,20]. RNA levels are depicted relative to undifferentiated cells on day 0 (n=2, error bars +/-s.e.m).

To determine whether *HERV-K(HML-2)* expression in pluripotent stem cells changes during differentiation, we induced embryoid body formation of hFib2-iPS5 cells. We applied semi-qRT-PCR analysis using the primer pair K7/K21 (see Figure 1B). Interestingly, the levels of *HERV-K(HML-2)* RNA started to decrease from day 6 onwards in hFib2-iPS5 cells, concomitant with a decrease of *NANOG* and an increase of *LMNA* expression (Figure 2A). To estimate the degree of the changes we quantified the signal intensities of the RT-PCR bands visualized by ethidium bromide gel analysis (Figure 2A) relative to *ACTB* (see Additional file 1 “Materials and Methods”). *HERV-K(HML-2)* expression levels dropped from 100% on day 0 to roughly 50% on day 6, and to 20% on day 10. Similarly, *NANOG* expression levels decreased from 100% on day 0 to roughly 75% on day 6, and 55% on day 10.

To verify this with a second method and cell line, we performed real-time RT-PCR analysis using differentiating H9 ESCs [19,20]. This analysis showed an even more dramatic reduction of *HERV-K(HML-2)* RNA expression during differentiation with significantly reduced transcription already detectable on day 2 of embryoid body differentiation (Figure 2B). Remarkably, the kinetics of *HERV-K(HML-2)* downregulation is even faster than repression of *OCT4* and *NANOG*.

These slightly divergent results may simply reflect the greater accuracy of real time PCR compared to semi-quantitive PCR or may be rooted in the well-known subtle differences in the expression profiles of pluripotent stem cells especially those of ESC compared to iPSC lines [24].

The recent findings that high RNA levels of the endogenous retrovirus group *HERV-H*[25] and expression of non-LTR retrotransposons [26] are also associated with human pluripotent stem cells indicate that a pluripotent phenotype might be accompanied by a general relieve of retroelement silencing. We therefore asked whether re-activation of *HERV-K(HML-2)* proviruses is specific for certain proviral elements or occurs in a stochastic manner.

### *HERV-K(HML-2)* proviruses are activated in ESC/iPSC/ECC lines in varying patterns

We analyzed activation of *HERV-K(HML-2)* proviruses in malignant and non-malignant pluripotent stem cells using an established protocol [27] to amplify, clone, and sequence a highly conserved part of the *gag* gene (see Additional file 1 “Materials and Methods”). A proviral sequence was scored as the genomic origin of the respective clone when the two sequences exerted more than 98% identity (Additional file 2 “Alignments”, .msf files of these alignments are available as Additional files 3 “K101(22q11.21)”, 4 “K10(5q33.3)”, 5 “K106(3q13.2)”, 6 “K115(8p23.1)”, 7 “K37(11q23.3)”, 8 “K102(1q22)”, 9 “K108(7p22.1)”, 10 “HKc10-B(10q24.2)”, 11 “K41(12q14.1)”, 12 “K109(6q14.1)” and 13 “KI(3q21.2)”). Out of the 66 full-length or nearly full-length *HERV-K(HML-2)* proviruses present in the human genome [1], a subset of 11 were found to be expressed in patterns, which varied from cell line to cell line (Table 1). More activated loci may be detected using optimized primers [28], deep sequencing techniques [29] as well as more cell lines. Notably, all transcribed loci belong to the LTR5Hs subgroup of *HERV-K(HML-2)* proviruses. Interestingly, phylogenetic classifications have shown that all human-specific retroviral integrations fall into this proviral subgroup [1]. Indeed, ten of the expressed proviruses were human-specific, while *K37(11q23.3)* may also be present in the genomes of great apes. Five of the re-activated proviruses belonged to type 1 and six to type 2 genotypes, respectively, but their number and expression levels varied between individual ESC/iPSC and ECC cell lines. Such variations in *HERV-K(HML-2)* proviral transcription profiles are commonly observed in somatic malignant and non-malignant tissues and cell lines [10,28] indicating a somewhat stochastic re-activation mechanism which may reflect varying chromatin dynamics [30]. By contrast, we observed constant and efficient transcription of the type 1 provirus *K101(22q11.21)* in all pluripotent stem cell lines tested, suggesting a more directed re-activation. *K101(22q11.21), K37(11q23.3)* and *K10(5q33.3)* encode *gag* and *protease* genes and may account for the protein expression detected, e.g. in the H9 and hFib2-iPS5 lines (Figure 1D). Transcription of one or the other type 2 locus was detected in the ESC/iPSC lines, but only at low level. This is in contrast to the observed constant up-regulation of *K108(7p22.1)* expression exclusively in the malignant ECC lines. Notably, a recent survey of *HERV-K(HML-2)* proviruses transcribed in melanomas in comparison to melanocytes also demonstrated *K108(7p22.1)* derived mRNA only in melanomas but not in the non-malignant precursor cells [28].

**Table 1:** Relative cloning frequencies of HERV-K(HML-2) loci transcribed in ESC, iPSC and ECC lines

			**ESC**	**ESC**	**ESC**	**iPSC**	**iPSC**	**iPSC**	**iPSC**	**iPSC**	**iPSC**	**ECC**	**ECC**	**ECC**
**Alias**	**Type**	**ORF**	**BG01**	**H1**	**H9**	**MRC**	**MRC**	**MSC**	**MSC**	**hFib2**	**hFib2**	**GH**	**NCCIT**	**2102**
						**iPS7**	**iPS20**	**iPS1**	**iPS3**	**iPS4**	**iPS5**			**Ep**
K101(22q11.21)	1	g/n	64	60	57	69	61	56	88	62	39	75	16	29
ERVK-24														
K10(5q33.3)	1	g/prt/n	-	26	7,3	8	23	5,5	6	-	22	8,5	21	-
ERVK-10														
K109(6q14.1)	2	g/prt/e/r	-	7	7,3	-	8	-	-	-	-	-	-	42
ERVK-9														
K106(3q13.2)	1	g/n	-	-	-	-	-	5,5	-	8	11	-	5	-
ERVK-3														
K37(11q23.3)	1	g/prt/n	36	-	14	15	8	22	-	15	16	-	-	-
ERVK-20														
HKc10-B(10q24.2)	2	g/r	-	7	7	-	-	11	-	-	6	-	-	-
ERVK-17														
KI(3q21.2)	2	r	-	-	-	8	-	-	6	-	6	-	-	-
ERVK-4														
K115(8p23.1)	2	pol/e/r	-	-	-	-	-	-	-	15	-	-	-	-
ERVK-8														
K41(12q14.1)	2	g/prt/e/r	-	-	7,3	-	-	-	-	-	-	-	-	-
ERVK-21														
K108(7p22.1)	2	g/prt/e/r	-	-	-	-	-	-	-	-	-	16,5	11	29
ERVK-6														
K102(1q22)	1	n	-	-	-	-	-	-	-	-	-	-	47	-
ERVK-7														

Proviruses are designated by name and chromosomal locus, by type and putative open reading frames (ORF). For each cell line the relative cloning frequencies of the respective HERV-K(HML-2) loci are given as % (number of sequences assigned to the locus divided by the total number of sequences generated from the cDNA sample). 14 clones were generated and analyzed for BG01, 2102Ep, H9; 15 for H1; 13 for MRCiPS7, MRCiPS20, hFib2iPS4; 17 for MSCiPS3; 18 for hFib2iPS5; 19 for MSCiPS1 and NCCIT, 24 for GH. Open reading frames for Gag (g), Protease (prt) Envelope (e), Rec (r) NP9 (n).

## Conclusion

The endogenous betaretrovirus group *HERV-K(HML-2)* is unique in its potential to code for viral and accessory proteins. We observed, in slightly varying patterns, re-activation at the RNA and protein levels of certain human specific, protein encoding *HERV-K(HML-2)* proviruses with promoter competent LTR sequences in pluripotent ESCs, iPSCs, and ECCs. The surprisingly constant activation of *K101(22q11.21)* across all cell lines tested may indicate a particular function in or association with pluripotency. We detected *K108(7p22.1)* only in the malignant ECC lines. This corresponds to up-regulated *K108(7p22.1)* transcription described in other germ cell tumours, in brain tumours but not in normal brain [10] as well as in malignant melanoma [28] but not in melanocytes. *HERV-K(HML-2)* proviruses were rapidly silenced upon embryoid body differentiation. Re-activation of this HERV group thus represents another marker for the undifferentiated state of pluripotent stem cells.

Active type 2 proviruses produce Rec protein, which supports cell transformation *in vitro* and germ line carcinoma *in situ* in transgenic mice [7,31]. Type 1 proviruses produce Np9 protein [4]. Both Rec and Np9 interact with transcriptional regulators. For example, Rec and Np9 bind the MYC repressor PLZF resulting in overexpression of MYC [6] and Np9 can bind to LNX [8] which might influence the activity of the NOTCH pathway. MYC and NOTCH are often involved in carcinogenesis but are also important players in the signalling networks controlling self-renewal, pluripotency and differentiation [32]. Since there are substantial differences between the cell cycle regulation of murine and human pluripotent stem cells [21], it is intriguing to speculate that Rec and Np9, which are not encoded by rodent ERVs, might play positive roles in human ESC/iPSCs. The observation that Rec and Np9 encoding *HERV-K(HML-2)* proviruses were preferentially activated in pluripotent stem cells and that their expression was simultaneously silenced upon differentiation independent of their chromosomal localization is in favour of such an idea.

## Competing interests

The authors declare that no competing interests exist.

## Authors’ contributions

NVF, SL and RL conceived the study and performed the analyses. NVF did *HERV-K(HML-2)* cloning, performed the sequence analysis and did the immunoblot assay. SL performed cell culture, embryoid body differentiation and RT-PCR analysis. GQD supplied the cells. ZI and GQD financially supported the study. JL helped interpret the results. NVF, SL, JL and RL wrote the manuscript. All authors have read and approved the submission of the manuscript.

## Supplementary Material

Additional file 1 “Materials and Methods”. Click here for file

Additional file 10 “HKc10-B(10q24.2)”. Click here for file

Additional file 11 “K41(12q14.1)”. Click here for file

Additional file 12 “K109(6q14.1)”. Click here for file

Additional file 13 “KI(3q21.2)”. Click here for file

Additional file 2 “Alignments”. Click here for file

Additional file 3 “K101(22q11.21)”. Click here for file

Additional file 4 “K10(5q33.3)”. Click here for file

Additional file 5 “K106(3q13.2)”. Click here for file

Additional file 6 “K115(8p23.1)”. Click here for file

Additional file 7 “K37(11q23.3)”. Click here for file

Additional file 8 “K102(1q22)”. Click here for file

Additional file 9 “K108(7p22.1)”. Click here for file
