# Supplementary material for: Human endogenous retrovirus K (HML-2) RNA and protein expression is a marker for human embryonic and induced pluripotent stem cells
Source: Retrovirology. 2013 Oct 24;10:115. doi: 10.1186/1742-4690-10-115 (PMC3819666; doi:10.1186/1742-4690-10-115)
Supplement: Additional file 1 — “Materials and Methods”. [file 1742-4690-10-115-S1.doc]

Additional file 1

Materials and Methods

Cell culture, embryoid body (EB) differentiation and RT-PCR analysis of ESC and iPSC lines

Testicular cancer cell lines NCCIT, GH and 2102EP were grown as described [1]. Fibroblasts (hFib2, MSC, and MRC5), ES (H1, H9, BG01), and iPS (hFib2-iPS4, hFib2-iPS5, MSC-iPS1, MSC-iPS3, MRC5-iPS5, MRC5-iPS20) cells were cultured and EB differentiation in suspension was performed as described in [2]. Work on human ESCs was approved by the Embryonic Stem Cell Research Oversight Committee of the Boston Children's Hospital, protocol numbers CHB SC 2005 8.24 and CHB SC 2006 8.17.

Total RNA was isolated using Trizol (Tel-Test) according to the manufacturer’s instructions and cDNA was generated from 1µg RNA with Superscript II (Invitrogen).

(Semi-quantitative) RT-PCR: Primer sequences to detect cellular *OCT4*, *NANOG*, *LMNA*, and *ACTB* were described before [2,3]. In Figure 2A an alternative *LMNA* forward primer (TGA AAG CGC GCA ATA CCA AGA AGG) was used resulting in a 354bp product. The following primers were used to detect *HERV-K(HML*-*2)* transcripts: K20: GAG GCT GGC GGG ATC CT and K6: CTA TCT CAG TAG ATG GAG CAT ACA AT as well as the primer pair K21 ATG AAC CCA TCA GAG ATG CAA AG and K7: TCT GTT TAA CAA AGC ACA TCC TGC was used. PCR conditions were as follows: K20/K6, K21/K7: 26-30 cycles, annealing temperature touchdown 58-55°C; *OCT4*, *NANOG*, *LMNA, ACTB*: 25 cycles, annealing temperature 60°C. The cycle numbers were chosen at a range within which amplification was still exponential and had not yet reached a plateau. To estimate the possible changes in the *HERV-K(HML-2)* and *NANOG* expression levels upon initiation of differentiation by embryoid body formation we quantified the signal intensities of the RT-PCR amplicons visualized in the ethidium bromid gel analysis shown in Figure 2A with the GelQuant-software from DNR Bio-Imaging System using *ACTB* as normalizer. The values were compared to those obtained for day 0 (undifferentiated cells) which were set as 100%.

Quantitative real time RT-PCR: Detection and primer sequences of cellular *OCT4*, *NANOG*, and *ACTB* have been described before [3]. Expression of *HERV-K(HML*-*2)* fragments *HERV-K(HML*-*2) rec*, *HERV-K(HML*-*2)* *NP9*, and *HERV-K(HML*-*2)* *env* was measured under identical conditions using the forward primer P_for_: CCA ACG TGG AGG CTT TTC TCTAG in combination with P_rev_REC: GGG TAT ACC TGC AGA CAC CAT TG, P_rev_NP9: GTA CAC CTG CAG TCT CCG TCT CC and P_rev_ENV: AGG GAG ACT TAC CAC CAT TGA TAC, respectively. cDNA was synthesized with Superscript II (Invitrogen) and qPCR was performed using the BrilliantSYBRGreenQPCRmix. Relative expression values were calculated (ΔΔCT method) using *ACTB* as normalizer.

Immunoblot

For immunoblot analysis 2,5 x 10^6^ cells were lysed as described in [4]. 30µg protein per lane were separated on 4-12 % Bis/Tris gels (Invitrogen) and transferred to nitrocellulose membrane (Schleicher und Schuell). Membranes were blocked over night at 4°C with blocking solution containing 4% milk powder in PBST [phosphate-buffered saline (PBS) with 0.05 % Tween20]. After washing with PBST the membranes were incubated first with the HERV-K(HML-2) Gag-specific monoclonal antibodies HERMA6/7 [5] (hybridoma supernatant, undiluted) for 1h at room temperature and afterwards consecutively with OCT4 antibody (Santa Cruz 1:750) and ACTB antibody (1:20 000 Sigma). Peroxidase coupled secondary antibodies (donkey anti-mouse, Amersham, dilution 1:10.000,) were incubated for 1h at room temperature. All antibodies were diluted in PBST with 2 % milk powder. Antibody binding was visualized using the chemiluminescence detection solution ECL (Amersham).

*HERV-K(HML-2)* *gag* cloning and identification of reactivated HERV-K(HML-2) loci

Isolation of cytoplasmic RNA was performed with the RNAeasy Midi-Kit (Qiagen), for poly-A RNA isolation we used the PolyATract-Kit (Roche)cDNA was generated from 1µg RNA with Superscript II (Invitrogen). .*HERV-K(HML-2)* *gag* sequences were amplified by PCR, cloned, sequenced and analysed using the method and the primer pair gag+ and gag- (see Figure 1B) published [6,7]. In detail, each PCR experiment contained 30pmol primers and 5µl c-DNA. Amplifications were done with AmpliTaq (Applied Biosystems) in a total volume of 50µl with following cycle conditions: one cycle 94°C for 5min; 35 cycles 95°C for 30s, 58°C for 30s, 72°C for 40s; 1 cycle 72°C for 10min. PCR products were excised from agarose gels, purified (MinElute, Qiagen) and ligated into the pGEM-T Easy vector (Promega). Plasmids from randomly selected transformed DH5α clones were purified with a QIAprep miniprep kit (Qiagen) and sequenced by MWG-Eurofins. Sequences were analysed using BLAT (http://genome.ucsc.edu/ (GRCh37/hg19)) and the Vector NTI-Software (Invitrogen). Proviral loci showing more than 98% identity to the respective database sequence were supposed to be the genomic origin of the *HERV-K(HML*-2) transcripts analysed. Sequences not matching to a reference proviral sequence were dismissed because they most probably correspond to recombined cDNAs due to the experimental set up [8]. We assumed that the frequencies with which we detected distinct *HERV-K(HML*-2) sequences within the total number of clones analyzed reflects the relative transcription rate of the respective provirus.

Reference List

1. Bieda K, Hoffmann A, Boller K: Phenotypic heterogeneity of human endogenous retrovirus particles produced by teratocarcinoma cell lines. *J Gen Virol* 2001, 82: 591-596.

2. Park IH, Zhao R, West JA, Yabuuchi A, Huo H, Ince TA *et al*.: Reprogramming of human somatic cells to pluripotency with defined factors. *Nature* 2008, 451: 141-146.

3. Loewer S, Cabili MN, Guttman M, Loh YH, Thomas K, Park IH *et al*.: Large intergenic non-coding RNA-RoR modulates reprogramming of human induced pluripotent stem cells. *Nat Genet* 2010, 42: 1113-1117.

4. Fuchs NV, Kraft M, Tondera C, Hanschmann KM, Lower J, Lower R: Expression of the human endogenous retrovirus group HML-2/HERV-K does not depend on canonical promoter elements but is regulated by the transcription factors Sp1 and Sp3. *J Virol* 2011.

5. Boller K, Schonfeld K, Lischer S, Fischer N, Hoffmann A, Kurth R *et al*.: Human endogenous retrovirus HERV-K113 is capable of producing intact viral particles. *J Gen Virol* 2008, 89: 567-572.

6. Flockerzi A, Ruggieri A, Frank O, Sauter M, Maldener E, Kopper B *et al*.: Expression patterns of transcribed human endogenous retrovirus HERV-K(HML-2) loci in human tissues and the need for a HERV Transcriptome Project. *BMC Genomics* 2008, 9: 354.

7. Ruprecht K, Ferreira H, Flockerzi A, Wahl S, Sauter M, Mayer J *et al*.: Human endogenous retrovirus family HERV-K(HML-2) RNA transcripts are selectively packaged into retroviral particles produced by the human germ cell tumor line Tera-1 and originate mainly from a provirus on chromosome 22q11.21. *J Virol* 2008, 82: 10008-10016.

8. Flockerzi A, Maydt J, Frank O, Ruggieri A, Maldener E, Seifarth W *et al*.: Expression pattern analysis of transcribed HERV sequences is complicated by ex vivo recombination. *Retrovirology* 2007, 4: 39.
