# Supplementary material for: Human endogenous retrovirus K (HML-2) RNA and protein expression is a marker for human embryonic and induced pluripotent stem cells
Source: Retrovirology. 2013 Oct 24;10:115. doi: 10.1186/1742-4690-10-115 (PMC3819666; doi:10.1186/1742-4690-10-115)
Supplement: Additional file 2 — “Alignments”. [file 1742-4690-10-115-S2.pdf]

```
                       *        20         *        40         *        60         *        80         *       100         *       120         *       140         *
              GCAGCCCTATTTCTTCGGACCTGTTCTTGTACCCCATCAATCCACCAAGTCTTAAATTGTAAAAATTGAGAGGGTGAGAGAGACGATTTTGCCAGAATCTCCCAATCATAAGGAATGAGTCTATGTCCATAAGCAATGGAATCTAATAA
HK-101      : ..................................................................................................................................................... : 149
BGO1_2      : ..................................................................................................................................................... : 149
BG01_3      : ..................................................................................................................................................... : 149
BG01_4      : ..................................................................................................................................................... : 149
BG01_5      : ..................................................................................................................................................... : 149
BG01_6      : ..................................................................................................................................................... : 149
BG01_7      : ..................................................................................................................................................... : 149
BG01_8      : ..................................................................................................................................................... : 149
BG01_9      : ..................................................................................................................................................... : 149
BG01_10     : ..................................................................................................................................................... : 149
H1_1        : ..................................................................................................................................................... : 149
H1_4        : ..................................................................................................................................................... : 149
H1_5        : ..................................................................................................................................................... : 149
H1_6        : ..................................................................................................................................................... : 149
H1_9        : ..................................................................................................................................................... : 149
H1_10       : ..................................................................................................................................................... : 149
H1_11       : ..................................................................................................................................................... : 149
H1_12       : ..................................................................................................................................................... : 149
H1_13       : ..................................................................................................................................................... : 149
H9_1        : ..................................................................................................................................................... : 149
H9_2        : ..................................................................................................................................................... : 149
H9_3        : ..................................................................................................................................................... : 149
H9_4        : .........G........................................................................................................................................... : 149
```

```
H9_5        : ..................................................................................................................................................... : 149
H9_6        : ..................................................................................................................................................... : 149
H9_7        : ................................................................................G.................................................................... : 149
H9_10       : ..................................................................................................................................................... : 149
MRCiPS7_1   : C.................................................................................................................................................... : 149
MRCiPS7_2   : ..................................................................................................................................................... : 149
MRCiPS7_4   : ..................................................................................................................................................... : 149
MRCiPS7_5   : ..................................................................................................................................................... : 149
MRCiPS7_6   : C.................................................................................................................................................... : 149
MRCiPS7_7   : C.................................................................................................................................................... : 149
MRCiPS7_10 : ..................................................................................................................................................... : 149
MRCiPS7_12 : ..............................G...................................................................................................................... : 149
MRCiPS7_13 : ..................................................................................................................................................... : 149
MRCiPS20_1 : ..................................................................................................................................................... : 149
MRCiPS20_5 : ..................................................................................................................................................... : 149
MRCiPS20_6 : ..................................................................................................................................................... : 149
MRCiPS20_7 : ..................................................................................................................................................... : 149
MRCiPS20_8 : ..................................................................................................................................................... : 149
MRCiPS20_9 : ..................................................................................................................................................... : 149
MRCiPS20_1 : ...C..T...........................................T.................................................................................................. : 149
MRCiPS20_1 : ..................................................................................................................................................... : 149
MSCiPS1_1   : ..................................................................................................................................................... : 149
MSCiPS1_3   : ..................................................................................................................................................... : 149
MSCiPS1_5   : ................................T...........................................................................................................T........ : 149
MSCiPS1_7   : ..................................................................................................................................................... : 149
```

```
MSCiPS1_8  : ..................................................................................................................................................... : 149
MSCiPS1_10 : ..................................................................................G.................................................................. : 149
MSCiPS1_11 : ..................................................................................................................................................... : 149
MSCiPS1_12 : ..................................T.................................................................................................................. : 149
MSCiPS1_15 : .............................C....................................................................................................................... : 149
MSCiPS1_16 : ..................................................................................................................................................... : 149
MSCiPS1_19 : ..................................................................................................................................................... : 149
MSCiPS3_1  : ..................................................................................................................................................... : 149
MSCiPS3_3  : ..................................................................................................................................................... : 149
MSCiPS3_4  : ..................................................................................................................................................... : 149
MSCiPS3_5  : ..................................................................................................................................................... : 149
MSCiPS3_6  : ..................................................................................................................................................... : 149
MSCiPS3_7  : ..................................................................................................................................................... : 149
MSCiPS3_8  : ..................................................................................................................................................... : 149
MSCiPS3_9  : ...................................................................................T.........................................C....G.................. : 149
MSCiPS3_11 : ..................................................................................................................................................... : 149
MSCiPS3_12 : .........-........................................................................................................................................... : 148
MSCiPS3_13 : ..................................................................................................................................................... : 149
MSCiPS3_14 : ..................................................................................................................................................... : 149
MSCiPS3_15 : .....................................................................................................................C............................... : 149
MSCiPS3_16 : ..................................................................................................................................................... : 149
MSCiPS3_17 : ..................................................................................................................................................... : 149
hFibiPS4_2 : ..................................................................................................................................................... : 149
hFibiPS4_4 : ..................................................................................................................................................... : 149
hFibiPS4_5 : ..................................................................................................................................................... : 149
```

```
hFibiPS4_6 : .........................................................................................................C........................................... : 149
hFibiPS4_8 : ..................................................................................................................................................... : 149
hFibiPS4_1 : ..................................................................................................................................................... : 149
hFibiPS4_1 : ..................................................................................................................................................... : 149
hFibiPS4_1 : ..................................................................................................................................................... : 149
hFibiPS5_3 : ..................................................................................................................................................... : 149
hFibiPS5_6 : ..................................................................................................................................................... : 149
hFibiPS5_7 : ..................................................................................................................................................... : 149
hFibiPS5_8 : ..................................................................................................................................................... : 149
hFibiPS5_1 : ..................................................................................................................................................... : 149
hFibiPS5_1 : ..................................................................................................................................................... : 149
GH_2       : ..................................................................................................................................................... : 149
GH_3       : ..................................................................................................................................................... : 148
GH_4       : ..................................................................................................................................................... : 148
GH_5       : .....................................T............................................................................................G.................. : 148
GH_9       : ..................................................................................................................................................... : 148
GH_10      : ..................................................................................................................................................... : 148
GH_13      : ..................................................................................................................................................... : 148
GH_14      : ..................................................................................................................................................... : 149
GH_15      : ..................................................................................................................................................... : 149
GH_16      : ..................................................................................................................................................... : 149
GH_17      : ..................................................................................................................................................... : 148
GH_18      : ..................................................................................................................................................... : 149
GH_19      :...................................................................................................................................G.................. : 150
GH_20      : ..................................................................................................................................................... : 149
```

```
GH_21       : ..................................................................................................................................................... : 149
GH_22       : ...........................C..................................................................................................................... : 149
GH_23       : ..................................................................................................................................................... : 149
GH_24       : ..................................................................................................................................................... : 149
NCCIT_1     : ..................................................................................................................................................... : 148
NCCIT_13    : ..................................................................................................................................................... : 148
NCCIT_16    : .....................................T............................................................................................G.................. : 148
2102EP_2    : ..........................................................................................................................G.................G........ : 149
2102EP_4    : ..................................................................................................................................................... : 149
2102EP_11   : ..........................................................................................................................G.......................... : 149
2102EP_6    : ..........................................................................................................................G.......................... : 149

                  160         *        180         *        200         *        220         *        240         *        260         *        280         *        300
             TGTCCTCATATAAGGGGAGTTGGGTCCATACTGTTTTACTCCCTCTTTCATATCTTTTAGCATTTT TATCGAAAAAGACTTGTATCTGGCCTCAACTGTGGGAGGCTCTCCCTCTTGGGCTCCTTCTCCAGGTGGCATCGGTTCTAACG
HK-101      : ....................................................................-.................................................................................... : 298
BG01_2      : ....................................................................-.................................................................................... : 298
BG01_3      : ....................................................................-.................................................................................... : 298
BG01_4      : ....................................................................-.................................................................................... : 298
BG01_5      : ....................................................................-.................................................................................... : 298
BG01_6      : ....................................................................-.................................................................................... : 298
BG01_7      : ....................................................................-.................................................................................... : 298
BG01_8      : ....................................................................-.................................................................................... : 298
BG01_9      : ....................................................................-.................................................................................... : 298
BG01_10     : ....................................................................-.................................................................................... : 298
H1_1        : ....................................................................-.................................................................................... : 298
```

```
H1_4       : ..................................................................-................................................................................... : 298
H1_5       : ..................................................................-................................................................................... : 298
H1_6       : ..................................................................-................................................................................... : 298
H1_9       : ..................................................................-................................................................................... : 298
H1_10      : ..................................................................-................................................................................... : 298
H1_11      : ..................................................................-................................................................................... : 298
H1_12      : ................................................................C.-................................................................................... : 298
H1_13      : ..................................................................-................................................................................... : 298
H9_1       : ..................................................................-................................................................................... : 298
H9_2       : ..................................................................-................................................................................... : 298
H9_3       : ..................................................................-................................................................................... : 298
H9_4       : ..................................................................-................................................................................... : 298
H9_5       : ..................................................................-................................................................................... : 298
H9_6       : ..................................................................-................................................................................... : 298
H9_7       : ..................................................................-................................................................................... : 298
H9_10      : ..................................................................-................................................................................... : 298
MRCiPS7_1  : ..................................................................-................................................................................... : 298
MRCiPS7_2  : ..................................................................-................................................................................... : 298
MRCiPS7_4  : ..................................................................-................................................................................... : 298
MRCiPS7_5  : ..................................................................-................................................................................... : 298
MRCiPS7_6  : ..................................................................-................................................................................... : 298
MRCiPS7_7  : ..................................................................-................................................................................... : 298
MRCiPS7_10 : ..................................................................-................................................................................... : 298
MRCiPS7_12 : ..................................................................-................................................................................... : 298
MRCiPS7_13 : ..................................................................-................................................................................... : 298
```

```
MRCiPS20_1 : ..............................................................-.............................................................................. : 298
MRCiPS20_5 : ..............................................................-.............................................................................. : 298
MRCiPS20_6 : ..............................................................-.............................................................................. : 298
MRCiPS20_7 : ..............................................................-.............................................................................. : 298
MRCiPS20_8 : ..............................................................-.............................................................................. : 298
MRCiPS20_9 : ..............................................................-.............................................................................. : 298
MRCiPS20_1 : ..............................................................-.............................................................................. : 298
MRCiPS20_1 : ..............................................................-.............................................................................. : 298
MSCiPS1_1  : ..............................................................-.............................................................................. : 298
MSCiPS1_3  : ..............................................................-.............................................................................. : 298
MSCiPS1_5  : ..............................................................-....................................A......................................... : 298
MSCiPS1_7  : ..............................................................-.............................................................................. : 298
MSCiPS1_8  : .....C.....................................C..................-.............................................................................. : 298
MSCiPS1_10 : ..............................................................-.......G...................................................................... : 298
MSCiPS1_11 : ..............................................................-.............................................................................. : 298
MSCiPS1_12 : ..............................................................-.............................................................................. : 298
MSCiPS1_15 : ..............................................................-.............................................................................. : 298
MSCiPS1_16 : ..............................................................-.............................................................................. : 298
MSCiPS1_19 : ..............................................................-.............................................................................. : 298
MSCiPS3_1  : ..............................................................-.............................................................................. : 298
MSCiPS3_3  : ..............................................................-.............................................................................. : 298
MSCiPS3_4  : ..............................................................-.............................................................................. : 298
MSCiPS3_5  : ..............................................................-.............................................................................. : 298
MSCiPS3_6  : ..............................................................-.............................................................................. : 298
MSCiPS3_7  : ..............................................................-.............................................................................. : 298
```

```
MSCiPS3_8  : ..................................................................-................................................................................... : 298
MSCiPS3_9  : ................A.................................................-...A............................................................................... : 298
MSCiPS3_11 : ..................................................................-................................................................................... : 298
MSCiPS3_12 : ..................................................................-................................................................................... : 297
MSCiPS3_13 : ..................................................................-................................................................................... : 298
MSCiPS3_14 : ..................................................................-................................................................................... : 298
MSCiPS3_15 : ..................................................................-................................................................................... : 298
MSCiPS3_16 : ..................................................................-................................................................................... : 298
MSCiPS3_17 : ..................................................................-................................................................................... : 298
hFibiPS4_2 : ..................................................................-................................................................................... : 298
hFibiPS4_4 : ..................................................................-................................................................................... : 298
hFibiPS4_5 : ..................................................................-................................................................................... : 298
hFibiPS4_6 : ..................................................................-................................................................................... : 298
hFibiPS4_8 : ..................................................................-................................................................................... : 298
hFibiPS4_1 : ..................................................................-................................................................................... : 298
hFibiPS4_1 : ..................................................................-................................................................................... : 298
hFibiPS4_1 : ..................................................................-................................................................................... : 298
hFibiPS5_3 : ..................................................................-................................................................................... : 298
hFibiPS5_6 : ..................................................................-................................................................................... : 298
hFibiPS5_7 : ..................................................................-................................................................................... : 298
hFibiPS5_8 : ..................................................................-................................................................................... : 298
hFibiPS5_1 : ..................................................................-................................................................................... : 298
hFibiPS5_1 : ..................................................................-................................................................................... : 298
GH_2       : ..................................................................-................................................................................... : 298
GH_3       : ......................................................C...........-................................................................................... : 297
```

GH_4 : ....-... : 297

GH_5 : ....T.. : 298

GH_9 : ....-... : 297

GH_10 : ....-...........................G... : 297

GH_13 : ....C......................-..................A.. : 297

GH_14 : ....-... : 298

GH_15 : ....-... : 298

GH_16 : ....-... : 298

GH_17 : ....-... : 297

GH_18 : ....-... : 298

GH_19 : ....-... : 299

GH_20 : ....-... : 298

GH_21 : .....C..-... : 298

GH_22 : ....-... : 298

GH_23 : ....-... : 298

GH_24 : ....-... : 298

NCCIT_1 : ....-... : 297

NCCIT_13 : ....-... : 297

NCCIT_16 : ....T.. : 298

2102EP_2 : ....-... : 298

2102EP_4 : ....-... : 298

2102EP_11 : ....-... : 298

2102EP_6 : ....-... : 298

```
                      *       320         *       340         *       360         *       380         *       400         *       420         *       440         *
             TTACTGGGAATTGCCATG CTCAGTATCTCCTTCCTTTCTTGATTTATCAATAATTTCATGTAATTCACTACCCTGTCTACTAGGTGGTGCCGTAGGATTAAGTCTCCTAGTGGGCGGCTGAGGGTATGGCGCCCTGCCCTGTGGTGCTG
HK-101     : ...................................................................................................................................................... : 448
BGO1_2     : ...................................................................................................................................................... : 448
BG01_3     : ...................................................................................................................................................... : 448
BG01_4     : ..................T................................................................................................................................... : 448
BG01_5     : .............................................................................................................................C........................ : 448
BG01_6     : ................................................T..................................................................................................... : 448
BG01_7     : ...................................................................................................................................................... : 448
BG01_8     : ...................................................................................................................................................... : 448
BG01_9     : ...................................................................................................................................................... : 448
BG01_10    : .......................................................A.............................................................................................. : 448
H1_1       : ........................................................................---------.........................................A........................... : 439
H1_4       : ...................................................................................................................................................... : 448
H1_5       : ...................................................................................................................................................... : 448
H1_6       : ...................................................................................................................................................... : 448
H1_9       : ........................................................................T............................................................................. : 448
H1_10      : ...................................................................................................................................................... : 448
H1_11      : ..............................T....................................................................................................................... : 448
H1_12      : ...................................................................................................................................................... : 448
H1_13      : ...................................................................................................................................................... : 448
H9_1       : ..................T................................................................................................................................... : 448
H9_2       : ...................................................................................................................................................... : 448
H9_3       : ..................T................................................................................................................................... : 448
H9_4       : ..................T.......C........................................................................................................................... : 448
```

```
H9_5        : ...................................................................................................................................................... : 448
H9_6        : ..................T....................................................................................................................................... : 448
H9_7        : ...................................................................................................................................................... : 448
H9_10       : ...................................................................................................................................................... : 448
MRCiPS7_1   : ...................................................................................................................................................... : 448
MRCiPS7_2   : ...................................................................................................................................................... : 448
MRCiPS7_4   : ...................................................................................................................................................... : 448
MRCiPS7_5   : ..................T....................................................................................................................................... : 448
MRCiPS7_6   : ...................................................................................................................................................... : 448
MRCiPS7_7   : ...................................................................................................................................................... : 448
MRCiPS7_10 : .................................................G.................................................................................................... : 448
MRCiPS7_12 : ..................T....................................................................................................................................... : 448
MRCiPS7_13 : .C................T....................................................................................................................................... : 448
MRCiPS20_1 : ..................T....................................................................................................................................... : 448
MRCiPS20_5 : ...................................................................................................................................................... : 448
MRCiPS20_6 : ..................T....................................................................................................................................... : 448
MRCiPS20_7 : ..................T....................................................................................................................................... : 448
MRCiPS20_8 : .......................................C.............................................................................................................. : 448
MRCiPS20_9 : ...................................................................................................................................................... : 448
MRCiPS20_1 : .....A............T....................................................................................................................................... : 448
MRCiPS20_1 : ..................T.......................................................................................................T........................... : 448
MSCiPS1_1   : ....................................................................................................................A................................. : 448
MSCiPS1_3   : ...................................................................................................................................................... : 448
MSCiPS1_5   : ...................................................................................................................................................... : 448
MSCiPS1_7   : ..................T.......................................................C........................................................................... : 448
```

```
MSCiPS1_8  : ..................T....................................................................................................................................... : 448
MSCiPS1_10 : ............................C......................................................................................................................... : 448
MSCiPS1_11 : ....................................................................................................................A................................. : 448
MSCiPS1_12 : ...................................................................................................................................................... : 448
MSCiPS1_15 : ..................T....................................................................................................................................... : 448
MSCiPS1_16 : ..................T....................................................................................................................................... : 448
MSCiPS1_19 : ...................................................................................................................................................... : 448
MSCiPS3_1  : ...................................................................................................................................................... : 448
MSCiPS3_3  : ..................T....................................................................................................................................... : 448
MSCiPS3_4  : ..................T....................................................................................................................................... : 448
MSCiPS3_5  : ..................T....................................................................................................................................... : 448
MSCiPS3_6  : ..................T....................................................................................................................................... : 448
MSCiPS3_7  : ..................T....................................................................................................................................... : 448
MSCiPS3_8  : ...................................................................................................................................................... : 448
MSCiPS3_9  : .........................................................................................................................G............................ : 448
MSCiPS3_11 : ..................T....................................................................................................................................... : 448
MSCiPS3_12 : ..................T....................................................................................................................................... : 447
MSCiPS3_13 : ..................T....................................................................................................................................... : 448
MSCiPS3_14 : ...................................................................................................................................................... : 448
MSCiPS3_15 : ..................T....................................................................................................................................... : 448
MSCiPS3_16 : ..................T....................................................................................................................................... : 448
MSCiPS3_17 : ...................................................................................................................................................... : 448
hFibiPS4_2 : ..................T....................................................................................................................................... : 448
hFibiPS4_4 : ...................................................................................................................................................... : 448
hFibiPS4_5 : ...................................................................................................................................................... : 448
```

```
hFibiPS4_6 : ..................T....................................................................................................................................... : 448
hFibiPS4_8 : ...................................................................................................................................................... : 448
hFibiPS4_1 : ...................................................................................................................................................... : 448
hFibiPS4_1 : ...................................................................................................................................................... : 448
hFibiPS4_1 : ................C..................................................................................................................................... : 448
hFibiPS5_3 : ..................T.....C............................................................................................................................. : 448
hFibiPS5_6 : ...................................................................................................................................................... : 448
hFibiPS5_7 : ..................T....................................................................................................................................... : 448
hFibiPS5_8 : ..................T....................................................................................................................................... : 448
hFibiPS5_1 : ...................................................................................................................................................... : 448
hFibiPS5_1 : ...................................................................................................................................................... : 448
GH_2       : ...................................................................................................................................................... : 448
GH_3       : ...................................................................................................................................................... : 447
GH_4       : ...................................................................................................................................................... : 447
GH_5       : ...................................................................................................................................................... : 448
GH_9       : .....................................................................................................G....................T........................... : 447
GH_10      : ...................................................................................................................................................... : 447
GH_13      : ...................................................................................................................................................... : 447
GH_14      : ...................................................................................................................................................... : 448
GH_15      : ...................................................................................................................................................... : 448
GH_16      : ...................................................................................................................................................... : 448
GH_17      : ...................................................................................................................................................... : 447
GH_18      : ....................................................................................................G................................................. : 448
GH_19      : ...................................................................................................................................................... : 449
GH_20      : ...................................................................................................................................................... : 448
```

```
GH_21      : ...................................................................................................................................................... : 448
GH_22      : ...................................................................................................................................................... : 448
GH_23      : ...................................................................................................................................................... : 448
GH_24      : ...................................................................................................................................................... : 448
NCCIT_1    : ...................................................................................................................................................... : 447
NCCIT_13   : ...................................................................................................................................................... : 447
NCCIT_16   : ...................................................................................................................................................... : 448
2102EP_2   : ...................................................................................................................................................... : 448
2102EP_4   : ...............G..................................................T................................................................................... : 448
2102EP_11  : ...........................................C.......................................................................................................... : 448
2102EP_6   : ...................................................................................................................................................... : 448

                    460         *       480         *       500         *       520         *       540         *       560         *       580         *       600
             GGGGCATTCCTGGATATCCATACTGACTTTCTGGGGGTGGCCGATACTGAAGTTCAGCCGGAGGCCAGTATTGATAGGCTACTGGCGGTTGGGTCTTATTTTCTTTAACCTGCTTTTGAGGTTGTAATGTTACGGGCACCTGACCTGCTG
HK-101     : ...................................................................................................................................................... : 598
BG01_2     : .....................................................................................................................................T................ : 598
BG01_3     : ...................................................................................................................................................... : 598
BG01_4     : ...................................................................................................................................................... : 598
BG01_5     : ...................................................................................................................................................... : 598
BG01_6     : ...................................................................................................................................................... : 598
BG01_7     : ...................................................................................................................................................... : 598
BG01_8     : ...................................................................................................................................................... : 598
BG01_9     : ...................................................................................................................................................... : 598
BG01_10    : ...................................................................................................................................................... : 598
H1_1       : ...................................................................................................................................................... : 589
```

```
H1_4       : ...................................................................................................................................................... : 598
H1_5       : .............................-........................................................................................................................ : 597
H1_6       : ...................................................................................................................................................... : 598
H1_9       : ...................................................................................................................................................... : 598
H1_10      : ...................................................................................................................................................... : 598
H1_11      : ...................................................................................................................................................... : 598
H1_12      : ...................................................................................................................................................... : 598
H1_13      : .......................C.............................................................................................................................. : 598
H9_1       : ...................................................................................................................................................... : 598
H9_2       : ...................................................................................................................................................... : 598
H9_3       : ...................................................................................................................................................... : 598
H9_4       : ...................................................................................................................................................... : 598
H9_5       : ...................................................................................................................................................... : 598
H9_6       : ...................................................................................................................................................... : 598
H9_7       : ...................................................................................................................................................... : 598
H9_10      : ...................................................................................................................................................... : 598
MRCiPS7_1  : ...................................................................................................................................................... : 598
MRCiPS7_2  : ...................................................................................................................................................... : 598
MRCiPS7_4  : ...................................................................................................................................................... : 598
MRCiPS7_5  : ...................................................................................................................................................... : 598
MRCiPS7_6  : ...................................................................................................................................................... : 598
MRCiPS7_7  : ...................................................................................................................................................... : 598
MRCiPS7_10 : ...................................................................................................................................................... : 598
MRCiPS7_12 : ...................................................................................................................................................... : 598
MRCiPS7_13 : ...................................................................................................................................................... : 598
```

```
MRCiPS20_1 : ...................................................................................................................................................... : 598
MRCiPS20_5 : .........................................................G.............................................................................................. : 598
MRCiPS20_6 : ...................................................................................................................................................... : 598
MRCiPS20_7 : ...................................................................................................................................................... : 598
MRCiPS20_8 : ...................................................................................................................................................... : 598
MRCiPS20_9 : ...................................................................................................................................................... : 598
MRCiPS20_1 : ...................................................................................................................................................... : 598
MRCiPS20_1 : ...............................................................................C...................................................................... : 598
MSCiPS1_1  : ...................................................................................................................................................... : 598
MSCiPS1_3  : .......C.............................................................................................................................................. : 598
MSCiPS1_5  : ...................................................................................................................................................... : 598
MSCiPS1_7  : ...................................................................................................................................................... : 598
MSCiPS1_8  : ...................................................................................................................................................... : 598
MSCiPS1_10 : ....................................................C................................................................................................. : 598
MSCiPS1_11 : ...................................................................................................................................................... : 598
MSCiPS1_12 : ...................................................................................................................................................... : 598
MSCiPS1_15 : ...................................................................................................................................................... : 598
MSCiPS1_16 : ...................................................................................................................................................... : 598
MSCiPS1_19 : ...................................................................................................................................................... : 598
MSCiPS3_1  : ...................................................................................................................................................... : 598
MSCiPS3_3  : ...................................................................................................................................................... : 598
MSCiPS3_4  : ...................................................................................................................................................... : 598
MSCiPS3_5  : ...................................................................................................................................................... : 598
MSCiPS3_6  : ...................................................................................................................................................... : 598
MSCiPS3_7  : ...................................................................................................................................................... : 598
```

```
MSCiPS3_8  : ...................................................................................................................................................... : 598
MSCiPS3_9  : ...................................................................................................................................................... : 598
MSCiPS3_11 : ...................................................................................................................................................... : 598
MSCiPS3_12 : ..........................................................................................................................C........................... : 597
MSCiPS3_13 : ...................................................................................................................................................... : 598
MSCiPS3_14 : ...................................................................................................................................................... : 598
MSCiPS3_15 : ...................................................................................................................................................... : 598
MSCiPS3_16 : ...................................................................................................................................................... : 598
MSCiPS3_17 : ...................................................................................................................................................... : 598
hFibiPS4_2 : ...................................................................................................................................................... : 598
hFibiPS4_4 : ...................................................................................................................................................... : 598
hFibiPS4_5 : ...................................................................................................................................................... : 598
hFibiPS4_6 : ...................................................................................................................................................... : 598
hFibiPS4_8 : ...................................................................................................................................................... : 598
hFibiPS4_1 : ...................................................................................................................................................... : 598
hFibiPS4_1 : ...................................................................................................................................................... : 598
hFibiPS4_1 : ...................................................................................................................................................... : 598
hFibiPS5_3 : ...................................................................................................................................................... : 598
hFibiPS5_6 : ...................................................................................................................................................... : 598
hFibiPS5_7 : ..............................................................................................................................C....................... : 598
hFibiPS5_8 : ..............................................................................................................................C....................... : 598
hFibiPS5_1 : ...................................................................................................................................................... : 598
hFibiPS5_1 : ...................................................................................................................................................... : 598
GH_2       : ...................................................................................................................................................... : 598
GH_3       : .......................................................................................................C........................C.....A............... : 597
```

```
GH_4       : ...................................................................................................................................................... : 597
GH_5       : ..................................................G................................................................................................... : 598
GH_9       : .......................C.............................................................................................................................. : 597
GH_13      : ...........................C.......................................................T............................................C.....A............... : 597
GH_14      : ...................................................................................................................................................... : 598
GH_15      : ...................................................................................................................................................... : 598
GH_16      : ...................................................................................................................................................... : 598
GH_17      : ...................................................................................................................................................... : 597
GH_18      : ...................................................................................................................................................... : 598
GH_19      : ...................................................................................................................................................... : 599
GH_20      : ...................................................................................................................................................... : 598
GH_21      : ...................................................................................................................................................... : 598
GH_22      : ...................................................................................................................................................... : 598
GH_23      : ...................................................................................................................................................... : 598
GH_24      : ...................................................................................................................................................... : 598
NCCIT_1    : ...................................................................................................................................................... : 597
NCCIT_13   : ...................................................................................................................................................... : 597
NCCIT_16   : ..................................................G................................................................................................... : 598
2102EP_2   : ...................................................................................................................................................... : 598
2102EP_4   : .....................................................................................................................................A................ : 598
2102EP_11  : ...................................................................................................................................................... : 598
2102EP_6   : .....G......................................................................................................................G........A................ : 598

                      *       620         *       640         *       660         *       680
```

```
               GAAGAGGACTTGTCCCTCGTGGTTTAGACTCTGATGGCC

HK-101      : .......................................------------------------------------------- : 637

BGO1_2      : .......................................------------------------------------------- : 637

BG01_3      : .......................................------------------------------------------- : 637

BG01_4      : .......................................------------------------------------------- : 637

BG01_5      : .......................................------------------------------------------- : 637

BG01_6      : .......................................------------------------------------------- : 637

BG01_7      : .......................................------------------------------------------- : 637

BG01_8      : .......................................------------------------------------------- : 637

BG01_9      : .......................................------------------------------------------- : 637

BG01_10     : .......................................------------------------------------------- : 637

H1_1        : .......................................------------------------------------------- : 628

H1_4        : .......................................------------------------------------------- : 637

H1_5        : .......................................------------------------------------------- : 636

H1_6        : .......................................------------------------------------------- : 637

H1_9        : .......................................------------------------------------------- : 637

H1_10       : .......................................------------------------------------------- : 637

H1_11       : .......................................------------------------------------------- : 637

H1_12       : .......................................------------------------------------------- : 637

H1_13       : .......................................------------------------------------------- : 637

H9_1        : .......................................------------------------------------------- : 637

H9_2        : .......................................------------------------------------------- : 637

H9_3        : .......................................------------------------------------------- : 637

H9_4        : .......................................------------------------------------------- : 637

H9_5        : .......................................------------------------------------------- : 637
```

```
H9_6        : .......................................------------------------------------------- : 637
H9_7        : .......................................------------------------------------------- : 637
H9_10       : .............................-.........------------------------------------------- : 636
MRCiPS7_1   : .......................................------------------------------------------- : 637
MRCiPS7_2   : .......................................------------------------------------------- : 637
MRCiPS7_4   : .......................................------------------------------------------- : 637
MRCiPS7_5   : .......................................------------------------------------------- : 637
MRCiPS7_6   : .......................................------------------------------------------- : 637
MRCiPS7_7   : .......................................------------------------------------------- : 629
MRCiPS7_10  : .......................................------------------------------------------- : 637
MRCiPS7_12  : .......................................------------------------------------------- : 637
MRCiPS7_13  : .......................................------------------------------------------- : 637
MRCiPS20_1  : .......................................------------------------------------------- : 637
MRCiPS20_5  : .......................................------------------------------------------- : 637
MRCiPS20_6  : .......................................------------------------------------------- : 637
MRCiPS20_7  : .......................................------------------------------------------- : 637
MRCiPS20_8  : .......................................------------------------------------------- : 637
MRCiPS20_9  : .......................................------------------------------------------- : 637
MRCiPS20_1  : .......................................------------------------------------------- : 637
MRCiPS20_1  : .............G.........................------------------------------------------- : 637
MSCiPS1_1   : .......................................------------------------------------------- : 637
MSCiPS1_3   : .......................................------------------------------------------- : 637
MSCiPS1_5   : .......................................------------------------------------------- : 637
MSCiPS1_7   : .......................................------------------------------------------- : 637
MSCiPS1_8   : .......................................------------------------------------------- : 637
```

```
MSCiPS1_10 : .....................................----------------------------------------- : 637
MSCiPS1_11 : .....................................----------------------------------------- : 637
MSCiPS1_12 : .....................................----------------------------------------- : 637
MSCiPS1_15 : .....................................----------------------------------------- : 637
MSCiPS1_16 : .....................................----------------------------------------- : 637
MSCiPS1_19 : .....................................----------------------------------------- : 637
MSCiPS3_1  : .....................................----------------------------------------- : 637
MSCiPS3_3  : .....................................----------------------------------------- : 637
MSCiPS3_4  : ....................................------------------------------------------ : 636
MSCiPS3_5  : .....................................----------------------------------------- : 637
MSCiPS3_6  : ....................................------------------------------------------ : 636
MSCiPS3_7  : ......................................---------------------------------------- : 638
MSCiPS3_8  : .....................................----------------------------------------- : 637
MSCiPS3_9  : .....................................----------------------------------------- : 637
MSCiPS3_11 : .....................................----------------------------------------- : 637
MSCiPS3_12 : .....................................----------------------------------------- : 636
MSCiPS3_13 : .....................................----------------------------------------- : 636
MSCiPS3_14 : .....................................----------------------------------------- : 637
MSCiPS3_15 : .....................................----------------------------------------- : 637
MSCiPS3_16 : .....................................----------------------------------------- : 636
MSCiPS3_17 : .........C...........................----------------------------------------- : 637
hFibiPS4_2 : .....................................----------------------------------------- : 637
hFibiPS4_4 : .....................................----------------------------------------- : 637
hFibiPS4_5 : .....................................----------------------------------------- : 637
hFibiPS4_6 : .....................................----------------------------------------- : 637
```

```
hFibiPS4_8 : ......................................-------------------------------------------- : 636
hFibiPS4_1 : .......................................------------------------------------------- : 637
hFibiPS4_1 : .......................................------------------------------------------- : 637
hFibiPS4_1 : .......................................------------------------------------------- : 637
hFibiPS5_3 : .............G.........................------------------------------------------- : 637
hFibiPS5_6 : .......................................------------------------------------------- : 637
hFibiPS5_7 : .......................................------------------------------------------- : 637
hFibiPS5_8 : .......................................------------------------------------------- : 637
hFibiPS5_1 : .......................................------------------------------------------- : 637
hFibiPS5_1 : .......................................------------------------------------------- : 637
GH_2       : .......................................------------------------------------------- : 637
GH_3       : .............G.........................------------------------------------------- : 636
GH_4       : .......................................------------------------------------------- : 636
GH_5       : ..................................G.C.-------------------------------------------- : 636
GH_9       : .......................................------------------------------------------- : 636
GH_10      : .......................................------------------------------------------- : 636
GH_13      : .............G.........................------------------------------------------- : 636
GH_14      : .....T.......G.........................------------------------------------------- : 637
GH_15      : .......................................------------------------------------------- : 637
GH_16      : .......................................------------------------------------------- : 637
GH_17      : .......................................------------------------------------------- : 636
GH_18      : .......................................------------------------------------------- : 637
GH_19      : ......................................-------------------------------------------- : 637
GH_20      : .......................................------------------------------------------- : 637
GH_21      : .......................................------------------------------------------- : 637
```

```
GH_22      : .......................................------------------------------------------- : 637
GH_23      : .......................................------------------------------------------- : 637
GH_24      : .......................................------------------------------------------- : 637
NCCIT_1    : .......................................------------------------------------------- : 636
NCCIT_13   : .......................................------------------------------------------- : 636
NCCIT_16   : ..................................-....------------------------------------------- : 636
2102EP_2   : .......................................------------------------------------------- : 637
2102EP_4   : .............G.........................------------------------------------------- : 637
2102EP_11  : .......................................------------------------------------------- : 637
2102EP_6   : .............G.........................------------------------------------------- : 637
```

```
                    *        20         *        40         *        60         *        80         *       100         *       120         *       140         *
             CAGCCCTATTTCTTCGGACCTGTTCTTGTACCCCATCAATCCACCAAGTCTTAAATTGTAAAAATTGAGAGGGTGAGAGAGACGATTTTGCCAGAATCTCCCAATCATAAGGAATGAGTCTATGTCCATAAGCAATGGAATCTAATAAT
HK_10      : -..................................................................................................................................................... : 149
H1_3       : ...................................................................................................................................................... : 150
H1_7       : ...................................................................................................................................................... : 150
H1_14      : ...................................................................................................................................................... : 150
H1_15      : ...................................................................................................................................................... : 150
H9_13      : ...................................................................................................................................................... : 150
MRCiPS7_9  : ...................................................................................................................................................... : 150
MRCiPS20_3 : .................................................G.................................................................................................... : 150
MRCiPS20_4 : ...................................................................................................................................................... : 150
MRCiPS20_1 : ......T............................................................................................................................................... : 150
MSCiPS1_18 : .................................................G.................................................................................................... : 150
MSCiPS3_10 : ...................................................................................................................................................... : 150
hFibiPS5_1 : ...................................................................................................................................................... : 150
hFibiPS5_4 : ...................................................................................................................................................... : 150
hFibiPS5_9 : ...................................................................................................................................................... : 150
hFibiPS5_1 : ...................................................................................................................................................... : 150
GH_6       : -..................................................................................................................................................... : 149
GH_7       : -....................................................................................G................................................................ : 149
GH_8       : -....................................................................................G................................................................ : 149
GH_12      : -..................................................................................................................................................... : 149
NCCIT_9    : -..................................................................................................................................................... : 149
NCCIT_14   : -..................................................................................................................................................... : 149
NCCIT_15   : --.................................................................................................................................................... : 148
```

```
NCCIT_18   : -..................................................................................................................................................... : 149

                    160         *       180         *       200         *       220          *       240         *       260         *       280         *      300
             GTCCTCATATAAGGGGAGTTGGGTCCATACTGTTTTACTCCCTCTTTCATATCTTTTAGCATTTTTATCG AAAAAGACTTGTATCTGGCCTCAACTGTGGGAGGCTCTCCCTCTTGGGCTCCTTCTCCAGGTGGCATCGGTTCTAACGT
HK_10      : ......................................................................-............................................................................... : 298
H1_3       : ...................................................................A..-...............C.......................................CT...................... : 299
H1_7       : ......................................................................-............................................................................... : 299
H1_14      : ......................................................................-............................................................................... : 299
H1_15      : ......................................................................-............................................................................... : 299
H9_13      : ......................................................................-............................................................................... : 299
MRCiPS7_9  : ...................................................C..................-............................................................................... : 299
MRCiPS20_3 : ......................................................................-............................................................................... : 299
MRCiPS20_4 : ......................................................................-............................................................................... : 299
MRCiPS20_1 : ......................................................................-............................................................................... : 299
MSCiPS1_18 : ......................................................................-............................................................................... : 299
MSCiPS3_10 : ......................................................................-.........................................G..................................... : 299
hFibiPS5_1 : ......................................................................-............................................................................... : 299
hFibiPS5_4 : ......................................................................-............................................................................... : 299
hFibiPS5_9 : ......................................................................-............................................................................... : 299
hFibiPS5_1 : ......................................................................-.........................C..................................................... : 299
GH_6       : ......................................................................-............................................................................... : 298
GH_7       : ......................................................................-............................................................................... : 298
GH_8       : ......................................................................-............................................................................... : 298
GH_12      : ......................................................................-............................................................................... : 298
NCCIT_9    : ......................................................................-............................................................................... : 298
```

```
NCCIT_14   : ......................................................................-................................................................................ : 298
NCCIT_15   : ......................................................................-................................................................................ : 297
NCCIT_18   : ......................................................................-................................................................................ : 298

                     *       320         *       340         *       360         *       380         *       400         *       420         *       440         *
             TACTGGGAATTGCCATGCCTCAGTATCTCC TTCCTTTCTTGATTTATCAATAATTTCATGTAATTCACTACCCTGTCTACTAGGTGGTGCCATAGGATTAAGTCTCCTAGTGGGCGGCTGATGGTATGGCGCCCTGCCCTGTGGTGCTG
HK_10      : ..............................-........................................................................................................................ : 447
H1_3       : ...........T..CT..C...........-........................................................................................................................ : 448
H1_7       : ..............................-........................................................................................................................ : 448
H1_14      : ..............................-........................................................................................................................ : 448
H1_15      : ..............................-........................................................................................................................ : 448
H9_13      : ..............................-........................................................................................................................ : 448
MRCiPS7_9  : ..............................-........................................................................................................................ : 448
MRCiPS20_3 : ..............................-........................................................................................................................ : 448
MRCiPS20_4 : ..............................-........................................................................................................................ : 448
MRCiPS20_1 : ..............................-........................................................................................................................ : 448
MSCiPS1_18 : ..............................-........................................................................................................................ : 448
MSCiPS3_10 : ..............................-........................................................................................................................ : 448
hFibiPS5_1 : ..............................-........................................................................................................................ : 448
hFibiPS5_4 : ..............................-........................................................................................................................ : 448
hFibiPS5_9 : ..............................-........................................................................................................................ : 448
hFibiPS5_1 : ..............................-........................................................................................................................ : 448
GH_6       : ..............................-........................................................................................................................ : 447
GH_7       : ..............................-........................................................................................................................ : 447
GH_8       : ..............................-........................................................................................................................ : 447
```

```
GH_12       : ..............................-....................................................................................................................... : 447
NCCIT_9     : ..............................-....................................................................................................................... : 447
NCCIT_14    : ..............................-....................................................................................................................... : 447
NCCIT_15    : ..............................-....................................................................................................................... : 446
NCCIT_18    : ..............................-....................................................................................................................... : 447

                      460         *        480         *        500         *        520         *        540         *        560         *        580         *        600
              GGGGCATTCCTGGATATCCATACTGACTTTCTGGGGGTGGCCGATACTGAAGTTCAGCCAGCGGCCAGTATT GATAGGCTACTTGCGGTTGGGTCTTATTTT CTTTAACCTGCTTTTGAGGTTGTAATCTTACG GCACC TGACCTG
HK_10       : .................................................................-......-..............................-......................................-....... : 593
H1_3        : ........................................................................-..............................-..........................G.....G.....-....... : 595
H1_7        : ........................................................................-..............................-..........................G.....G.....-....... : 595
H1_14       : ........................................................................-..............................-......................................-....... : 595
H1_15       : ........................................................................-..............................-......................................-....... : 595
H9_13       : ........................................................................-..............................-T.....................................-....... : 595
MRCiPS7_9   : ........................................................................-..............................-......................................-....... : 595
MRCiPS20_3  : ........................................................................-..............................-......................................-....... : 595
MRCiPS20_4  : ...................................................................................................................................................... : 598
MRCiPS20_1  : ........................................................................-..............................-......................................-....... : 595
MSCiPS1_18  : ........................................................................-..............................-......................................-....... : 595
MSCiPS3_10  : ...........................................................G............-...........G..................-................................G.....-....... : 595
hFibiPS5_1  : ........................................................................-..............................-..............................T.......-....... : 595
hFibiPS5_4  : ........................................................................-..............................-......................................-....... : 595
hFibiPS5_9  : ........................................................................-..............................-......................................-....... : 595
hFibiPS5_1  : .....................................C..................................-..............................-......................................-....... : 595
GH_6        : ........................................................................-..............................-......................................-....... : 594
```

```
GH_7       : ........................................................................-..............................-..............A.......................-....... : 594
GH_8       : ........................................................................-..............................-..............A.......................-....... : 594
GH_12      : ........................................................................-..............................-......................................-....... : 594
NCCIT_9    : ...........................................................G............-...........G..................-................................G.....-....... : 594
NCCIT_14   : ...........................................................G............-...........G..................-................................G.....-....... : 594
NCCIT_15   : ...........................................................G............-...........G..................-................................G.....-....... : 593
NCCIT_18   : ...........................................................G............-...........G..................-................................G.....-....... : 594

                     *       620         *       640         *       660         *       680
             CTGGAAGAGGACTTGTGCCTCGTGGTTTAGACTCTGATGGCC
HK_10      : ..........................................-------------------------------------- : 635
H1_3       : ................C.........................-------------------------------------- : 637
H1_7       : ................C.........................-------------------------------------- : 637
H1_14      : ..........................................-------------------------------------- : 637
H1_15      : ..........................................-------------------------------------- : 637
H9_13      : ..........................................-------------------------------------- : 637
MRCiPS7_9  : ..............................-...........-------------------------------------- : 636
MRCiPS20_3 : ..........................................-------------------------------------- : 637
MRCiPS20_4 : ..........................................-------------------------------------- : 640
MRCiPS20_1 : ..........................................-------------------------------------- : 637
MSCiPS1_18 : ..........................................-------------------------------------- : 637
MSCiPS3_10 : ..........................................-------------------------------------- : 637
hFibiPS5_1 : ..........................................-------------------------------------- : 637
hFibiPS5_4 : ..........................................-------------------------------------- : 637
hFibiPS5_9 : ..........................................-------------------------------------- : 637
```

```
hFibiPS5_1 : ..........................................--------------------------------------- : 637

GH_6       : .......................................T..--------------------------------------- : 636

GH_7       : ..........................................--------------------------------------- : 636

GH_8       : ..........................................--------------------------------------- : 636

GH_12      : ..........................................--------------------------------------- : 636

NCCIT_9    : ..........................................--------------------------------------- : 636

NCCIT_14   : ..........................................--------------------------------------- : 636

NCCIT_15   : ..........................................--------------------------------------- : 635

NCCIT_18   : ..........................................--------------------------------------- : 636
```

```
                      *        20         *        40         *        60         *        80         *       100         *       120         *       140         *
             GCAGCCCTATTTCTTCGGACCTGTTCTTGTACCCCATCAATCCACCAAGTCTTAAATTGTAAAAATTGAGAGGGTGAGAGAGACGATTTTGCCAGAATCTCCCAATCATAAGGAATGAGTCTATGTCCATGAGCAATGGAATCTAATAAT
HK_109     : ...................................................................................................................................................... : 150
H1_2       : ...................................................................................................................................................... : 150
H9_12      : ...................................................................................................................................................... : 150
MRCiPS20_1 : ...................................................................................................................................................... : 150
2102EP_8   : ...................................................................................................................................................... : 150
2102EP_10  : ..........................................T........................................................................................................... : 150
2102EP_12  : ..........................................................................................................................G........................... : 150
2102EP_13  : ...........................................................................................................................................G.......... : 150
2102EP_14  : ...................................................................................................................................................... : 150
2102EP_7   : ......................................................................................................A............................................... : 150

                    160         *       180         *       200         *       220         *       240         *       260         *       280         *       300
             GTCCTCATATAAGGGGAGTTGGGTCCATACTGTTTTACTCCCTCTTTCATATCTTTTAGTATTTTTATCGAAAAAGACTTGTATCTGGCCTCAACTGTGGGAGGCTCTCCCTCTTGGGCTCCTTCTCCAGGTGGCATCGGTTCTAACGTT
HK_109     : ...................................................................................................................................................... : 300
H1_2       : ...................................................................................................................................................... : 300
H9_12      : ...................................................................................................................................................... : 300
MRCiPS20_1 : ...................................................................................................................................................... : 300
2102EP_8   : ...................................................................................................................................................... : 300
2102EP_10  : .............................................................................................................................C........................ : 300
2102EP_12  : ...................................................................................................................................................... : 300
2102EP_13  : ...................................................................................................................................................... : 300
2102EP_14  : ...........................................................C.......................................................................................... : 300
```

```
2102EP_7   : ...................................................................................................................................................... : 300

                    *        320         *        340         *        360         *        380         *        400         *        420         *        440         *
             ACTGGGAATTGCCATGCCTCAGTATCTCCTTCCTTTCTTGATTTATCAATAATTTCATGTAATTCACTACCCTGTCTACTAGGTGGTGCCGTAGGATTAAGTCTCCTAGTGGGCGGCTGAGGGTATGGCGCCCTGCCCTGTGGTGCTGGG
HK_109     : ...................................................................................................................................................... : 450
H1_2       : .............................................................................................--....................................................... : 448
H9_12      : ...................................................................................................................................................... : 450
MRCiPS20_1 : ...................................................................................................................................................... : 450
2102EP_8   : ...................................................................................................................................................... : 450
2102EP_10  : ...................................................................................................................................................... : 450
2102EP_12  : ...................................................................................................................................................... : 450
2102EP_13  : ...................................................................................................................................................... : 450
2102EP_14  : .....................................................................................................................C................................ : 450
2102EP_7   : ...AA................................................................................................................................................. : 450

                   460         *        480         *        500         *        520         *        540         *        560         *        580         *        600
             GGCATTCCTGGATATCCATACTGACTTTCTGGGGGTGGCCGATACTGAAGTTCAGCCGGAGGCCAGTATTGATAGGCTACTGGCGGTTGGGTCTTATTTTCTTTAACCTGCTTTTGAGGTTGTAATGTTACAGGCACCTGACCTGCTGGA
HK_109     : ...................................................................................................................................................... : 600
H1_2       : ...................................................................................................................................................... : 598
H9_12      : ...................................................................................................................................................... : 600
MRCiPS20_1 : ...................................-.................................................................................................................. : 599
2102EP_8   : ...................................................................................................................................G.................. : 600
2102EP_10  : ...................................................................................................................................................... : 600
2102EP_12  : ...................................................................................................................................................... : 600
2102EP_13  : .........................................................A.C.....................T............................................C....GA................. : 600
```

```
2102EP_14  : .....C................................................................................................................................................ : 600
2102EP_7   : ...................................................................................................................................................... : 600

                     *       620         *       640         *       660         *
             AGAGGACTTGTGCCTCGTGGTTTAGACTCTGATGGCC
HK_109     : .....................................----------------------------------------- : 637
H1_2       : .....................................----------------------------------------- : 635
H9_12      : .....................................----------------------------------------- : 637
MRCiPS20_1 : .....................................----------------------------------------- : 636
2102EP_8   : .....................................----------------------------------------- : 637
2102EP_10  : ...........C.........................----------------------------------------- : 637
2102EP_12  : .....................................----------------------------------------- : 637
2102EP_13  : .....................................----------------------------------------- : 637
2102EP_14  : .....................................----------------------------------------- : 637
2102EP_7   : .....................................----------------------------------------- : 637
```

```
                      *        20         *        40         *        60         *        80         *       100         *       120         *       140         *
             GCAGCCCTATTTCTTCGGACCTGTTCTTGTACCCCATCAATCCACCAAGTCTTAAATTGTAAAAATTGAGAGGGTGAGAGAGACGATTTTGCCAGAATCTCCCAATCATAAGGAATGAGTCTATGTCCATGAGCAATGGAATCTAATAAT
HK_106     : ........................................................................................................................T............................. : 150
MSCiPS1_9  : ...................................................................................................................................................... : 150
hFibiPS4_1 : ...................................................................................................................................................... : 150
hFibiPS5_5 : ...................................................................................................................................................... : 150
hFibiPS5_1 : ..................................................................................................................................A................... : 150
NCCIT_22   : ...................................................................................................................................................... : 150

                    160         *       180         *       200         *       220         *       240         *       260         *       280         *       300
             GTCCTCATATAAGGGGAGTTGGGTCCATACTGTTTTACTCCCTCTTTCATATCTTTTAGCATTTTTATCGAAAAAGACTTGTATCTGGCCTCAACTGTGGGAGGCTCTCCCTCTTGGGCTCCTTCTCCAGGTGGCATCGGTTCTAACGTT
HK_106     : ...................................................................................................................................................... : 300
MSCiPS1_9  : ...................................................................................................................................................... : 300
hFibiPS4_1 : ...................................................................................................................................................... : 300
hFibiPS5_5 : ...................................................................................................................................................... : 300
hFibiPS5_1 : ...................................................................................................................................................... : 300
NCCIT_22   : ...................................................................................................................................................... : 300

                      *       320         *       340         *       360         *       380         *       400         *       420         *       440         *
             ACTGGGAATTGCCATGCCTCAGTATCTCCTTCCTTTCTTGATTTATCAATAATTTCATGTAATTCACTACCCCGTCTACTAGGTGGTGCCGTAGGATTAAGTCTCCTAGTGGGTGGCTGAGGGTATGGCGCCCTGCCCTGTGGTGCTGGG
HK_106     : ...................................................................................................................................................... : 450
MSCiPS1_9  : ...................................................................................................................................................... : 450
hFibiPS4_1 : ...................................................................................................................................................... : 450
hFibiPS5_5 : ...................................................................................................................................................... : 450
hFibiPS5_1 : ...................................................................................................................................................... : 450
```

```
NCCIT_22   : ...................................................................................................................................................... : 450

                    460         *       480         *       500         *       520         *       540         *       560         *       580         *       600
             GGCATTCCTGGATATCCATACTGACTTTCTGGGGGTGGCCGATACTGAAGTTCAGCCGG GGCCAGTATTGATAGGCTACTGGCGGTTGGGTCTTATTTTCTTTAACCTGCTTTTGAGGTTGTAATGTTACGGGCACCTGACCTGCTGGA
HK_106     : ...................................................................................................................................................... : 600
MSCiPS1_9  : ...................................................................................................................................................... : 600
hFibiPS4_1 : ...................................................................................................................................................... : 600
hFibiPS5_5 : ...........................................................T.......................................................................................... : 600
hFibiPS5_1 : ...........................................................T.......................................................................................... : 600
NCCIT_22   : ...................................................................................................................................................... : 600

                      *       620         *       640         *       660         *
             AGATGACTTGTGCCTCGTGGTTTAGACTCTGATGGCC
HK_106     : .....................................----------------------------------------- : 637
MSCiPS1_9  : .....................................----------------------------------------- : 637
hFibiPS4_1 : .....................................----------------------------------------- : 637
hFibiPS5_5 : .....................................----------------------------------------- : 637
hFibiPS5_1 : .....................................----------------------------------------- : 637
NCCIT_22   : .....................................----------------------------------------- : 637
```

```
                         *        20         *        40         *        60         *        80         *       100         *       120         *       140         *
                 GCAGCCCTATTTCTTCGGACCTGTTCTTGTACCCCATCAATCCACCAAGTCTTAAATTGTAAAAATTGAGAGGGTGAGAGAGATGATCTTGCCAGAATCTCCCAATCATAAGGAATGA GTCTATGTCC ATGAGCAATGGAATCTAATA
HK_37        : ......................................................................................................................-..........-.................... : 148
BGO1_1       : ......................................................................................................................-..........-.................... : 148
BG01_11      : ......................................................................................................................-..........-.................... : 148
BG01_12      : ......................................................................................................................-..........-.................... : 148
BG01_13      : ......................................................................................................................-..........-.................... : 148
BG01_14      : ......................................................................................................................-..........-.................... : 148
H9_9         : ......................................................................................................................-..........-.................... : 148
H9_14        : .........................................................................................C............................-..........-.................... : 148
MRCiPS7_3    : ......................................................................................................................-..........-.................... : 148
MRCiPS7_8    : ......................................................................................................................-..........-.................... : 148
MRCiPS20_2   : ......................................................................................................................-..........-.................... : 148
MSCiPS1_2    : ......................................................................................................................-..........-.................... : 148
MSCiPS1_4    : .......................................................................................T..............................------.....-.................... : 143
MSCiPS1_14   : ..........................................................................................................................C...AG.....-.-...CT..--....G : 146
MSCiPS1_17   : -......................................................................................T......................................A......-.-...CT..--....G : 145
hFibiPS4_3   : ...............................................................G......................................................-..........-.................... : 148
hFibiPS4_7   : ...............................................................G......................................................-..........-.................... : 148
hFibiPS5_2   : ......................................................................................................................-..........-.........A.......... : 148
hFibiPS5_1   : ......................................................................................................................-..........-.................... : 148

                     160         *       180         *       200         *       220         *       240         *       260         *       280         *       300
                 ATGTACTCATATAAGGGGAGTTGGGTCCATACTGTTTTACTCCCTCTTTCATATCTTTTAGCATTTTTATCAAAAAAGACTTGTATCTGGCCTCAACTGTGAGAGGCTCTCCCTCTTGGGCTTCTTCTCCAGGTGGCATCGGTTCTAACG
```

```
HK_37       : ...................................................................................................................................................... : 298
BGO1_1      : ...................................................................................................................................................... : 298
BG01_11     : .................................................................................................................C.................................... : 298
BG01_12     : ...................................................................................................................................................... : 298
BG01_13     : ...................................................................................................................................................... : 298
BG01_14     : ...................................................................................................................................................... : 298
H9_9        : ...................................................................................................................................................... : 298
H9_14       : ...................................................................................................................................................... : 298
MRCiPS7_3   : ...................................................................................................................................................... : 298
MRCiPS7_8   : ...................................................................................................................................................... : 298
MRCiPS20_2  : ...................................................................................................................................................... : 298
MSCiPS1_2   : ...................................................................................................................................................... : 298
MSCiPS1_4   : ...................................................................................................................................................... : 293
MSCiPS1_14 : ---................................................................................................................................................... : 293
MSCiPS1_17 : ---.................G.......................................T................A.....A.................................................................. : 292
hFibiPS4_3 : ...................................................................................................................................................... : 298
hFibiPS4_7 : ...................................................................................................................................................... : 298
hFibiPS5_2 : ...................................................................................................................................................... : 298
hFibiPS5_1 : ...................................................................................................................................................... : 298

                        *       320         *       340         *       360         *       380         *       400         *       420         *       440         *
              TTACTGGGAATTGCCACGCCTCAGTATCTCCTTCCTTTCTTCTCAATAATTTCATGTAATTCACTACCCTGTCTACTAGGCGGTGCCGTAGGATTAAGTCTCCTAGTGGGCGGCTGAGGGTATGGCGCCCTGCCCTGTGGTGCTGGGGGC
HK_37       : ...................................................................................................................................................... : 448
BGO1_1      : .........G....................................................................................A....................................................... : 448
BG01_11     : ...................................................................................................................................................... : 448
```

BG01_12 : .... : 448

BG01_13 : .... : 448

BG01_14 : .... : 448

H9_9 : .... : 448

H9_14 : ....-..... : 447

MRCiPS7_3 : ....-. : 447

MRCiPS7_8 : ....-.. : 447

MRCiPS20_2 : ....-. : 447

MSCiPS1_2 : ....-..... : 447

MSCiPS1_4 : .... : 443

MSCiPS1_14 : .... : 443

MSCiPS1_17 : .............A..A....................... : 442

hFibiPS4_3 : .... : 448

hFibiPS4_7 : .... : 448

hFibiPS5_2 : .... : 448

hFibiPS5_1 : .... : 448

460 * 480 * 500 * 520 * 540 * 560 * 580 * 600

ATTCCTGGATATCCATACTGACTTTCTGGGGGTGGCTGATACTGAAGTTCAGCCAGCGGCCAGTATTGATAAGCTACTGGTGGTTGGCTCTTATTTTCTCTAACCTGCGTTTGAGGTTGTAATGTTACGGGCACCTGACCTGCTGGAAGA

HK_37 : .... : 598

BGO1_1 : .... : 598

BG01_11 : ....T........... : 598

BG01_12 : .... : 598

BG01_13 : .... : 598

BG01_14 : .... : 598

H9_9 : .... : 598

H9_14 : .... : 597

MRCiPS7_3 : .... : 597

MRCiPS7_8 : .... : 597

MRCiPS20_2 : ....A..A.............................. : 597

MSCiPS1_2 : ....C... : 597

MSCiPS1_4 : .... : 593

MSCiPS1_14 : .... : 593

MSCiPS1_17 : ....C...C...........................A.....C. : 592

hFibiPS4_3 : ....................................C.................G.A..............G........C......G...........T........T... : 598

hFibiPS4_7 : ....................................C.................G.A..............G........C......G...........T........T... : 598

hFibiPS5_2 : .... : 598

hFibiPS5_1 : .... : 598

*   620   *   640   *   660   *   680

GGACTTGGCCCTCGTGGTTTAGACTCTGATGGCC

HK_37 : ....-- : 632

BGO1_1 : ....-- : 632

BG01_11 : ....-- : 632

BG01_12 : ....-- : 632

BG01_13 : ....-- : 632

BG01_14 : ....-- : 632

H9_9 : ....-- : 632

H9_14 : ....-- : 631

MRCiPS7_3 : ....-- : 631

```
MRCiPS7_8  : ..................................---------------------------------------------- : 631

MRCiPS20_2 : T....................-............---------------------------------------------- : 630

MSCiPS1_2  : ..................................---------------------------------------------- : 631

MSCiPS1_4  : .................................----------------------------------------------- : 626

MSCiPS1_14 : .................................----------------------------------------------- : 626

MSCiPS1_17 : .......A..........................---------------------------------------------- : 626

hFibiPS4_3 : .......T..........................---------------------------------------------- : 632

hFibiPS4_7 : .......T..........................---------------------------------------------- : 632

hFibiPS5_2 : ..................................---------------------------------------------- : 632

hFibiPS5_1 : ..................................---------------------------------------------- : 632
```

```
                   *         20          *         40          *         60          *         80          *        100          *        120          *        140          *
             GCAGCCCTATTTCTTCGGACCTGTTCTTGTACCCCATCAATCCACCAAGTCTTAAATTGTAAAAATTGAGAGGGTGAGAGAGACGATTTTGCCAGAATCTCCCAATCATAAGGAATGAGTCTATGTCCATGAGCAATGGAATCTAATAAT
ERVK-17    : ...................................................................................................................................................... : 150
H1_8       : ...................................................................................................................................................... : 150
H9_11      : ..................................................................................................................................A................... : 150
MSC_iPS_1_ : ........................C............................................................................................................................. : 150
MSC_iPS_1_ : ...................................................................................................................................................... : 150
hFib_iPS_5 : -...............................T.T................................................................................................................... : 149

                 160         *        180          *        200          *        220          *        240          *        260          *        280          *        300
             GTCCTCATATAAGGGGAGTTGGGTCCATACTGTTTTACTCCCTCTTTCATATCTTTTAGCATTTTTATCGAAAAAGACTTGTATCTGGCCTCAACTGTGGGAGGCTCTCCCTCTTGGGCTCCTTCTCCAGGTGGCATCGGTTCTAACGTT
ERVK-17    : .................T...................................................................................A.................................................. : 300
H1_8       : .........................................T.............................................................................................T.............. : 300
H9_11      : ...................................................................................................................................................... : 300
MSC_iPS_1_ : ................................C..................................................................................................................... : 300
MSC_iPS_1_ : ...................................................................................................................................................... : 300
hFib_iPS_5 : ..................................................................................................................................T...............T... : 299

                   *        320          *        340          *        360          *        380          *        400          *        420          *        440          *
             ACTGGGAATTGCCA GCCTCAGTATCTCCTTCCTTTCTTGATTTATCAATAATTTCATGTAATTCACTACCCTGTCTACTAGGTGGTGCCGTAGGATTAAGTCTCCTAGTGGG GGCTGAGGGTATGGCGCCCTGCCCTGTGGTGCTGGG
ERVK-17    : ..............C....................................................................................................................................... : 450
H1_8       : .................................................................................................................T.................................... : 450
H9_11      : ................T..................................................................................................................................... : 450
MSC_iPS_1_ : ...................................................................................................................................................... : 450
MSC_iPS_1_ : ........................................................................C........................................T.................................... : 450
```

```
hFib_iPS_5 : ..............C...........................................................................A.......................A................................... : 449

                    460         *       480         *       500         *       520         *       540         *       560         *       580         *       600
             GGCATTCCTGGATATCCATACTGACTTTCTGGGGGTGGCCGATACTGAAGTTCaGCcGG GGCCAGTATTGATA GCTaCTGGCGGTTGGGTCTTATTTTCTTTAACCTGC TTTGAGGTTGTAaT TTACGgGCACCTGACCTGCTGGA
ERVK-17    : .....................................................G..T......................................................G............G......................... : 600
H1_8       : ...............................................................................................................G..............C....................... : 600
H9_11      : ...........................................................A.......................................................................................... : 600
MSC_iPS_1_ : ...........................................................A.......................................................................................... : 600
MSC_iPS_1_ : ...................................................................................................................................................... : 600
hFib_iPS_5 : ..............................................................................T...............................................C.....A................. : 599

                      *       620         *       640         *       660         *
             AGA GACTTGT CCTCGTGGTTTAGACTCTGATGGCC
ERVK-17    : ..........GC........C................----------------------------------------- : 637
H1_8       : .....................................----------------------------------------- : 637
H9_11      : ...........C.........................----------------------------------------- : 637
MSC_iPS_1_ : ...T.................................----------------------------------------- : 637
MSC_iPS_1_ : ...T.................................----------------------------------------- : 637
hFib_iPS_5 : .......................-.............----------------------------------------- : 635
```

```
                        *        20         *        40         *        60         *        80         *       100         *       120         *       140         *
                GCAGCCCTATTTCTTCGGACCTGTTCTTGTACTCTATCAATCCACCAAGTCTTAAATTGTAAAAATTGAGAGGGTGAGAGAGACGATTTTGCCAGAATCTCCCAATCATAAGGAATGAGTCTATGTCCATGAGCAATGGAATCTAATAAT
HK_I        : ...................................................................................................................................................... : 150
MRCiPS7_11  : ...................................................................................................................................................... : 150
MSCiPS3_2   : ...................................................................................................................................................... : 150
hFbiPS5_18  : ...................................................................................................................................................... : 150

                      160         *       180         *       200         *       220         *       240         *       260         *       280         *       300
                GTCCTCATATAAGGGGAGTTGGGTCCATACTGTTTTACTCCCTCTTTCATATCTTTTAGCATTTTTATCGAAAAAGACTTGTATCTGGCCTCAACTGTGGGAGGCTCTCCCTCTTGGGCTCCTTCTCCAGTTGGCATCGGTTCTAATGTT
HK_I        : ...................................................................................................................................................... : 300
MRCiPS7_11  : ...................................................................................................................................................... : 300
MSCiPS3_2   : ...................................................................................................................................................... : 300
hFbiPS5_18  : ...................................................................................................................................................... : 300

                        *       320         *       340         *       360         *       380         *       400         *       420         *       440         *
                ACTGGGAATTGCCACGCCTCAGTATCTCCTTCCTTTCTTGATTTATCAATAATTTCATGTAATTCACTACCCTGTCTACTAGGTGGTGCCATAGGATTAAGTCTCCTAGTGGGCAGCTGAGGGTATGGCGCCCTGCCCTGTGGTGCTGGG
HK_I        : ...................................................................................................................................................... : 450
MRCiPS7_11  : ...................................................................................................................................................... : 450
MSCiPS3_2   : .....................................................................................A................................................................ : 450
hFbiPS5_18  : ...................................................................................................................................................... : 450

                      460         *       480         *       500         *       520         *       540         *       560         *       580         *       600
                GGCATTCCTGGATATCCATACTGACTTTCTGGGGGTGGCCGATACTGAAGTTCAGCCGGCGGCCAGTATTGATAAGCTTCTGGCGGTTGGGTCTTATTTTCTTTAACCTGC TTTGAGGTTGTAATGTTACAGGCACCTGACCTGCTGGA
HK_I        : ...................................................................................................................................................... : 600
MRCiPS7_11  : ...................................................................................................................................................... : 600
```

```
MSCiPS3_2  : ...............................................................................................................G...................................... : 600
hFbiPS5_18 : ...............................................................................................................T..............C....GA................. : 600

                      *       620         *       640         *       660         *
             AGAGGACTTGTGCCTCGTGGTTTAGACTCTGATGGCC
HK_I       : ................A....................----------------------------------------- : 637
MRCiPS7_11 : .....................................----------------------------------------- : 637
MSCiPS3_2  : .....................................----------------------------------------- : 637
hFbiPS5_18 : .......................-.............----------------------------------------- : 636
```

```
                      *        20         *        40         *        60         *        80         *       100         *       120         *       140         *
             GCAGCCCTATTTCTTCGGACCTGTTCTTGTACCCCATCAATCCACCAAGTCTTAAATTGTAAAAATTGAGAGGGTGAGAGAGACGATTTTGCCAGAATCTCCCAATCATAAGGAATGAGTCTATGTCCATGAGCAATGGAATCTAATAAT
HK_115     : ...................................................................................................................................................... : 150
hFibiPS4_1 : ...................................................................................................................................................... : 150
hFibiPS4_9 : ...................................................................................................................................................... : 150

                    160         *       180         *       200         *       220         *       240         *       260         *       280         *       300
             GTCCTCATATAAGGGGAGTTGGGTCCATACTGTTTTACTCCCTCTTTCATATCTTTTAGCATTTTTATCGAAAAAGACTTGTATCTGGCCTCAACTGTGGGAGGCTCTCCCTCTTGGGCTCCTTCTCCAGGTGGCATCGGTTCTAACGTT
HK_115     : ...................................................................................................................................................... : 300
hFibiPS4_1 : ...................................................................................................................................................... : 300
hFibiPS4_9 : ...................................................................................................................................................T.. : 300

                      *       320         *       340         *       360         *       380         *       400         *       420         *       440         *
             ACTGGGAATTGCCATGCCTCAGTATCTCCTTCCTTTCTTGATTTATCAATAATTTCATGTAATTCACTACCCTGTCTACTAGGTGGTGCCGTAGGATTAAGTCTCCTAGTGGGCGGCTGAGGGTATGGCTCCCTGCCCTGTGGTGCTGGG
HK_115     : ...................................................................................................................................................... : 450
hFibiPS4_1 : ...................................................................................................................................................... : 450
hFibiPS4_9 : ........................................................................C.......T............A......T............T..............T..................... : 450

                    460         *       480         *       500         *       520         *       540         *       560         *       580         *       600
             GGCATTCCTGGATATCCATACTGACTTTCTGGGGGTGGCCGATACTGAAGTTCAGCCGGAGGCCAGTATTGATAGGCTACTGGCGGTTGGGTCTTATTTTCTTTAACCTGCTTTTGAGGTTGTAATGTTACGGGCACCTGACCTGCTGGA
HK_115     : ...................................................................................................................................................... : 600
hFibiPS4_1 : ...................................................................................................................................................... : 600
hFibiPS4_9 : .......T.....T.....T........A......................................................................................................................... : 600
```

```
                     *        620         *        640         *        660         *
             AGAGGACTTGTCCCTCGTGGTTTAGACTCTGATGGCC
HK_115      : ...........G.........................: 637
hFibiPS4_1 : .....................................: 637
hFibiPS4_9 : ....................................-: 636
```

```
                   *        20         *        40         *        60         *        80         *       100         *       120         *       140         *
          GCAGCCCTATTTCTT GGACCTGTTCTTGTACCCCATCAATCCACCAAGTCTTAAATTGTAAAAATTGAGAGGGTAAGAGAGACGATTTTGCCAGAATCTCCCAATCATAAGGAATGAGTCTATGTCCATGAGCAATGGAATCTAATAAT
HK_41 : ...................................................................................................................................................... : 150
H9_8  : ...................................................................................................................................................... : 150

                160         *       180         *       200         *       220         *       240         *       260         *       280         *       300
          GTCCTCATATAAGGGGAGTTGGGTCCATACTGTTTTACTCCTTCTTTCATATCTTTTAGCATTTTTATTGAAAAAGACTTGTATCTGGCCTCAACTGTGGGAGGCTCTCCCTCTTGGGCTCCTTCTCCAGGTGGCATCGGTTCTAACATT
HK_41 : ...................................................................................................................................................... : 300
H9_8  : ...................................................................................................................................................... : 300

                   *       320         *       340         *       360         *       380         *       400         *       420         *       440         *
          ACTGGGAATTGCCATGCCTCAGTATCTCCTTCCTTTCTTGATTTATCAATAATTTCATGTAATTCACTACCCTGTCTACTAGGTGGTGCCGTAGGATTAAGTCTCCTAGTGGGCGGCTGAGGGTATGGCGCCCTGCCCTGTGGTGCTGGG
HK_41 : ...................................................................................................................................................... : 450
H9_8  : ...................................................................................................................................................... : 450

                460         *       480         *       500         *       520         *       540         *       560         *       580         *       600
          GGCATTCCTGGATATCCATACTGACTTTCTGGGGGTGGCCGATACTGAAGTTCAGCCGGCGGCCAGTATTGATAAGCTACTGGTGGTTGGGTCTTATTTTCTTTAACCTGCGTTTGAGGTTGTAATGTTACGGGCACCTGACCTGCTGGA
HK_41 : ...................................................................................................................................................... : 600
H9_8  : ...................................................................................................................................................... : 600

                   *       620         *       640         *       660         *
          AGAGGACTTGTGCCTCGTGGTTTAGACTCTGATGGCC
HK_41 : .....................................: 637
H9_8  : .....................................: 637
```

```
                  *        20         *        40         *        60         *        80         *       100         *       120         *       140         *
             GCAGCCCTATTTCTTCGGACCTGTTCTTGTACCCCATCAATCCACCAAGTCTTAAATTGTAAAAATTGAGAGGGTGAGAGAGACGATTTTGCCAGAATCTCCCAATCATAAGGAATGAGTCTATGTCCATGAGCAATGGAATCTAATAAT
HK_108     : ...................................................................................................................................................... : 150
GH_1       : ...................................................................................................................................................... : 150
GH_10      : ..................................................................................................................................A................... : 150
NCCIT_5    : ...................................................................................................................................................... : 150
NCCIT_17   : ...................................................................................................................................................... : 150
2102EP_1   : ...................................................................................................................................................... : 150
2102EP_3   : ...................................................................................................................................G.................. : 150
2102EP_5   : ...................................................................................................................................................... : 150
2102EP_9   : ...................................................................................................................................................... : 150

                160         *       180         *       200         *       220         *       240         *       260         *       280         *       300
             GTCCTCATATAAGGGGAGTTGGGTCCATACTGTTTTACTCCCTCTTTCATATCTTTTAGCTTTTTTATCGAAAAAGACTTGTATCTGGCCTCAACTGTGGGAGGCTCTCCCTCTTGGGCTCCTTCTCCAGGTGGCATCGGTTCTAACGTT
HK_108     : ...................................................................................................................................................... : 300
GH_1       : ...................................................................................................................................................... : 300
GH_10      : ............................................................A...............................G......................................................... : 300
NCCIT_5    : ............................................................A......................................................................................... : 300
NCCIT_17   : ...................................................................................................................................................... : 300
2102EP_1   : ...................................................................................................................................................... : 300
2102EP_3   : ...................................................................................................................................................... : 300
2102EP_5   : ...................................................................................................................................................... : 300
2102EP_9   : ...................................................................................................................................................... : 300

                  *       320         *       340         *       360         *       380         *       400         *       420         *       440         *
```

```
          ACTGGGAATTGCCATGCCTCAGTATCTCCTTCCTTTCTTGATTTATCAATAATT CATGTAATTTACTACCCTGTCTACTAGGTGGTGCCGTAGGATTAAGTCTCCTAGTGGGCGGCTGAGGGTATGGCGCCCTGCCCTGTGGTGCTGGG
HK_108   : ...................................................................................................................................................... : 450
GH_1     : .........................................................................................................................A............................ : 450
GH_10    : ................................................................C..................................................................................... : 450
NCCIT_5  : ....................G...........................................C.....................C............................................................... : 450
NCCIT_17 : .........................................................................................................................A............................ : 450
2102EP_1 : ......................................................A............................................................................................... : 450
2102EP_3 : ...................T.................................................................................................................................. : 450
2102EP_5 : ......................................................A............................................................................................... : 450
2102EP_9 : ......................................................A............................................................................................... : 450

                 460         *       480         *       500         *       520         *       540         *       560         *       580         *       600
          GGCATTCCTGGATATCCATACTGACTTTCTGGGGGTGGCCGATACTGAAGTTCAGCCGGAGGCCAGTATTGATAGGCTACTGGCGGTTGGGTCTTATTTTCTTTAACCTGCTTTTGAGGTTGTAATGTTACAGGCACCTGACCTGCTGGA
HK_108   : ...................................................................................................................................................... : 600
GH_1     : ...................................................................................................................................................... : 600
GH_10    : ...................................................................................................................................G.................. : 600
NCCIT_5  : ..............................................................................................................................C....G.................. : 600
NCCIT_17 : ...................................................................................................................................................... : 600
2102EP_1 : ...................................................................................................................................................... : 600
2102EP_3 : ...............................................................................................................................................A...... : 600
2102EP_5 : ...................................................................................................................................................... : 600
2102EP_9 : ...................................................................................................................................................... : 600

                 *       620         *       640         *       660         *       680         *
          AGAGGACTTGTGCCTCGTGGTTTAGACTCTGATGGCC
```

```
HK_108   : .....................................------------------------------------------------------------ : 637

GH_1     : .....................................------------------------------------------------------------ : 637

GH_10    : ...........C.........................------------------------------------------------------------ : 637

NCCIT_5  : .....................................------------------------------------------------------------ : 637

NCCIT_17 : .....................................------------------------------------------------------------ : 637

2102EP_1 : .....................................------------------------------------------------------------ : 637

2102EP_3 : .....................................------------------------------------------------------------ : 637

2102EP_5 : .....................................------------------------------------------------------------ : 637

2102EP_9 : .....................................------------------------------------------------------------ : 637
```

```
                   *        20         *        40         *        60         *        80         *       100         *       120         *       140         *
            CAGCCCTATTTCTTCGGACCTGTTCTTGTACCCCATCAATCCACCAAGTCTTAAATTGTAAAAATTGAGAGGGTGAGAGAGACGATTTTGCCAGAATCTCCCAATCATAAGGAATGAGTCTATGTCCAT AGCAATGGAATCTAATAAT
HK_102    : ...................................................................................................................................................... : 150
NCCIT_2   : -.................................................................................................................................A................... : 149
NCCIT_3   : ...................................................................................................................................................... : 150
NCCIT_4   : ...................................................................................................................................................... : 150
NCCIT_6   : -.......................................................................................................................................G............. : 149
NCCIT_7   : -..................................................................................................................................................... : 149
NCCIT_8   : -.................................................................................................................................A................... : 149
NCCIT_10  : -..................................................................................................................................................... : 149
NCCIT_11  : -...................................................................................T...C............................................................. : 149
NCCIT_12  : -.................................................................................................................................A................... : 149

                 160         *       180         *       200         *       220         *       240         *       260         *       280         *       300
            GTCCTCATATAAGGGGAGTTGGGTCCATACTGTTTTACTCCCTCTTTCATATCTTTTAGCATTTTTATCG AAAAAGACTTGTATCTGGCCTCAACTGTGGGAGGCTCTCCCTCTTGGGCTCCTTCTCCAGGTGGCATCGGTTCTAACGT
HK_102    : ......................................................................-............................................................................... : 299
NCCIT_2   : .................................................G....................-............................................................................... : 298
NCCIT_3   : ......................................................................-............................................................................... : 299
NCCIT_4   : ......................................................................-............................................................................... : 299
NCCIT_6   : ......................................................................-............................................................................... : 298
NCCIT_7   : ......................................................................-............................................................................... : 298
NCCIT_8   : .................................C.....................................--............................................................................. : 297
NCCIT_10  : ......................................................................-............................................................................... : 298
NCCIT_11  : ..A..................................................................-...............................A....................T........................... : 298
NCCIT_12  : ......................................................................-............................................................................... : 298
```

```
                   *       320         *       340         *       360         *       380         *       400         *       420         *       440         *
           TACTGGGAATTGCCATGCCTCAGTATCTCCTTCCTTTCTTGATTTATCAATAATTTCATGTAATTCACTACCCTGTCTACTAGGTGGTGCCGTAGGATTAAGTCTCCTAGTGGGCGGCTGAGGGTATGGCGCCCTGCCCTGTGGTGCTGG
HK_102   : ...................................................................................................................................................... : 449
NCCIT_2  : .................................................................T.................................................................................... : 448
NCCIT_3  : ...................................C...................................................C.............................................................. : 449
NCCIT_4  : ...................................................................................................................................................... : 449
NCCIT_6  : ...................C.................................................................................................................................. : 448
NCCIT_7  : ...................................................................................................................................................... : 448
NCCIT_8  : ......................................................C............................................................................................... : 447
NCCIT_10 : ...................................C...................................................C.............................................................. : 448
NCCIT_11 : ............................................................C..............................A.............................T............................ : 448
NCCIT_12 : ...........................................................................................A.............................T............C............... : 448

                 460         *       480         *       500         *       520         *       540         *       560         *       580         *       600
           GGGCATTCCTGGATATCCATACTGACTTTCTGGGGGTGGCCGATACTGAAGTTCAGCCGG GGCCAGTATTGATAGGCTACTGGCGGTTGGGTCTTATTTTCTTTAACCTGCTTTTGAGGTTGTAATGTTACGGGCACCTGACCTGCTGG
HK_102   : ...................................................................................................................................................... : 599
NCCIT_2  : ....................................................................................................................................A................. : 598
NCCIT_3  : ...................................................................................................................................................... : 599
NCCIT_4  : ...................................................................................................................................................... : 599
NCCIT_6  : .........................................................................................................................C............................ : 598
NCCIT_7  : ............................................................T......................................................................................... : 598
NCCIT_8  : ...................................................................................................................................................... : 597
NCCIT_10 : ...................................................................................................................................................... : 598
NCCIT_11 : ............................................................C..................................................................C...................... : 598
NCCIT_12 : ............................................................C..................................................................C..............-....... : 597
```

```
                    *       620         *       640         *       660         *       680
           AAGA GACTTGTGCCTCGTGGTTTAGACTCTGATGGCC
HK_102   : ......................................-------------------------------------------------- : 637
NCCIT_2  : ....G.................................-------------------------------------------------- : 636
NCCIT_3  : .............................................------------------------------------------- : 644
NCCIT_4  : .............................................------------------------------------------- : 644
NCCIT_6  : ......................................-------------------------------------------------- : 636
NCCIT_7  : ......................................-------------------------------------------------- : 636
NCCIT_8  : ......................................-------------------------------------------------- : 635
NCCIT_10 : ......................................-------------------------------------------------- : 636
NCCIT_11 : ....G...............................---------------------------------------------------- : 634
NCCIT_12 : ....G.................................-------------------------------------------------- : 635
```
